# A *Trypanosoma brucei* Kinesin Heavy Chain Promotes Parasite Growth by Triggering Host Arginase Activity

**DOI:** 10.1371/journal.ppat.1003731

**Published:** 2013-10-31

**Authors:** Géraldine De Muylder, Sylvie Daulouède, Laurence Lecordier, Pierrick Uzureau, Yannick Morias, Jan Van Den Abbeele, Guy Caljon, Michel Hérin, Philippe Holzmuller, Silla Semballa, Pierrette Courtois, Luc Vanhamme, Benoît Stijlemans, Patrick De Baetselier, Michael P. Barrett, Jillian L. Barlow, Andrew N. J. McKenzie, Luke Barron, Thomas A. Wynn, Alain Beschin, Philippe Vincendeau, Etienne Pays

**Affiliations:** 1 Laboratory of Molecular Parasitology, IBMM, Université Libre de Bruxelles (ULB), Gosselies, Belgium; 2 Laboratoire de Parasitologie, UMR 177 IRD CIRAD Université de Bordeaux, Bordeaux, France; 3 Myeloid Cell Immunology Laboratory, VIB Brussels, Brussels, Belgium; 4 Cellular and Molecular Immunology Unit, Vrije Universiteit Brussel (VUB), Brussels, Belgium; 5 Department of Biomedical Sciences, Veterinary Protozoology Unit, Prins Leopold Institute of Tropical Medicine Antwerp, Antwerp, Belgium; 6 Department of Pathology, Institut de Pathologie et de Génétique, Gosselies, Belgium; 7 The Wellcome Trust Centre for Molecular Parasitology, Institute for Infection, Immunity and Inflammation, College of Medical, Veterinary and Life Sciences, University of Glasgow, Glasgow, United Kingdom; 8 Glasgow Polyomics Facility, University of Glasgow, Glasgow, United Kingdom; 9 Laboratory of Molecular Biology, Medical Research Council, Cambridge, United Kingdom; 10 Immunopathogenesis Section, Laboratory of Parasitic Diseases, National Institute of Allergy and Infectious Diseases, National Institutes of Health, Bethesda, Maryland, United States of America; Washington University School of Medicine, United States of America

## Abstract

**Background:**

In order to promote infection, the blood-borne parasite *Trypanosoma brucei* releases factors that upregulate arginase expression and activity in myeloid cells.

**Methodology/Principal findings:**

By screening a cDNA library of *T. brucei* with an antibody neutralizing the arginase-inducing activity of parasite released factors, we identified a Kinesin Heavy Chain isoform, termed TbKHC1, as responsible for this effect. Following interaction with mouse myeloid cells, natural or recombinant TbKHC1 triggered SIGN-R1 receptor-dependent induction of IL-10 production, resulting in arginase-1 activation concomitant with reduction of nitric oxide (NO) synthase activity. This TbKHC1 activity was IL-4Rα-independent and did not mirror M2 activation of myeloid cells. As compared to wild-type *T. brucei*, infection by *TbKHC1* KO parasites was characterized by strongly reduced parasitaemia and prolonged host survival time. By treating infected mice with ornithine or with NO synthase inhibitor, we observed that during the first wave of parasitaemia the parasite growth-promoting effect of TbKHC1-mediated arginase activation resulted more from increased polyamine production than from reduction of NO synthesis. In late stage infection, TbKHC1-mediated reduction of NO synthesis appeared to contribute to liver damage linked to shortening of host survival time.

**Conclusion:**

A kinesin heavy chain released by *T. brucei* induces IL-10 and arginase-1 through SIGN-R1 signaling in myeloid cells, which promotes early trypanosome growth and favors parasite settlement in the host. Moreover, in the late stage of infection, the inhibition of NO synthesis by TbKHC1 contributes to liver pathogenicity.

## Author Summary

From the first invasive step onwards, African trypanosomes can efficiently undermine the protective immune response of their mammalian host to favor their survival within the host and successful transmission by its vector, the tsetse fly. Identifying the parasite factors affecting the protective immune response is thus critical to detail the immune evasion mechanisms of these organisms. We report here that during acute infection, a *Trypanosoma brucei* protein named Kinesin Heavy Chain 1 (TbKHC1) sustains the development of the first (most prominent) peak of parasitaemia in the blood and its control by the host. Mechanistically, TbKHC1 was found to interact with the SIGN-R1 molecule at the surface of immune cells. Hereby, TbKHC1 modulates arginine/NO metabolism in immune cells towards the production by the host of nutrients (polyamines) required for parasite growth via an IL-10-dependent induction of arginase 1 and down-regulation of iNOS activities. Consequently, IL-10/arginase 1 producing immune cells are impaired in their capacity to destroy the parasite, favouring parasite settlement. Moreover, in the late stage of infection, the inhibition of NO synthesis by TbKHC1 increases liver pathogenicity that contributes to compromised host survival. Thus, targeting TbKHC1 may benefit the host protective immunity against *T. brucei* parasite.

## Introduction

The protozoan flagellate parasite *Trypanosoma brucei* is responsible for the diseases human sleeping sickness and nagana in cattle. In experimental murine models, the host immune response to this blood-borne pathogen involves antibody production against the Variant Surface Glycoprotein (VSG), as well as interferon-γ (IFN-γ)-mediated activation of macrophages/myeloid cells into cells of the M1 phenotype. These engulf opsonized parasites and synthesize factors that interfere with trypanosome growth including tumor necrosis factor-α (TNF). However, uncontrolled IFN-γ-induced immune responses including TNF and NO production as the infection persists induce tissue pathogenicity and death of the host [1]–[5]. Induction of IL-10 can attenuate the IFN-γ/M1 response and hereby enables prolonged survival of *T. brucei*-infected mice [6]–[8].

Only few *T. brucei*-derived immunomodulatory factors have been identified. These include the glycosylphosphatidylinositol anchor of VSG, CpG oligodeoxynucleotides and the trypanosome suppressive immunomodulating factor (TSIF) which have been found to induce TNF- and NO-secreting M1 cells [9]–[12]. Factors inducing M1 cells favor the control of parasite development in the early stage of infection, but their continuous release in the late stage of infection can sustain inflammation responsible for tissue pathogenicity. Components found in the culture medium of *T. brucei* have also been shown to affect immune cells of the host. In particular, factors released by the parasite promote the degradation of L-arginine through increase of arginase activity in macrophages/myeloid cells, and antagonize NO synthases (NOS)-mediated conversion of L-arginine into NO in infected mice. Arginase induction appears to attenuate the innate response at the early stage of infection, and likely contributes to the synthesis of polyamines and the trypanosome anti-oxidant trypanothione, known to promote trypanosome growth and colonization of the host [5], [13]–[15]. We have identified TbKHC1, a kinesin heavy chain isoform, as a factor released by *T. brucei* to trigger host arginase-1 activity for promotion of its own growth in the host. Although arginase-1 expression is commonly associated with alternatively activated myeloid cell (M2) functions, TbKHC1-induced arginase-1 was independent of IL-4Rα signaling but relied on a SIGN-R1 receptor-dependent IL-10 secretion.

## Results

### A *T. brucei* kinesin heavy chain induces arginase activity in myeloid cells

**Figure 1:**
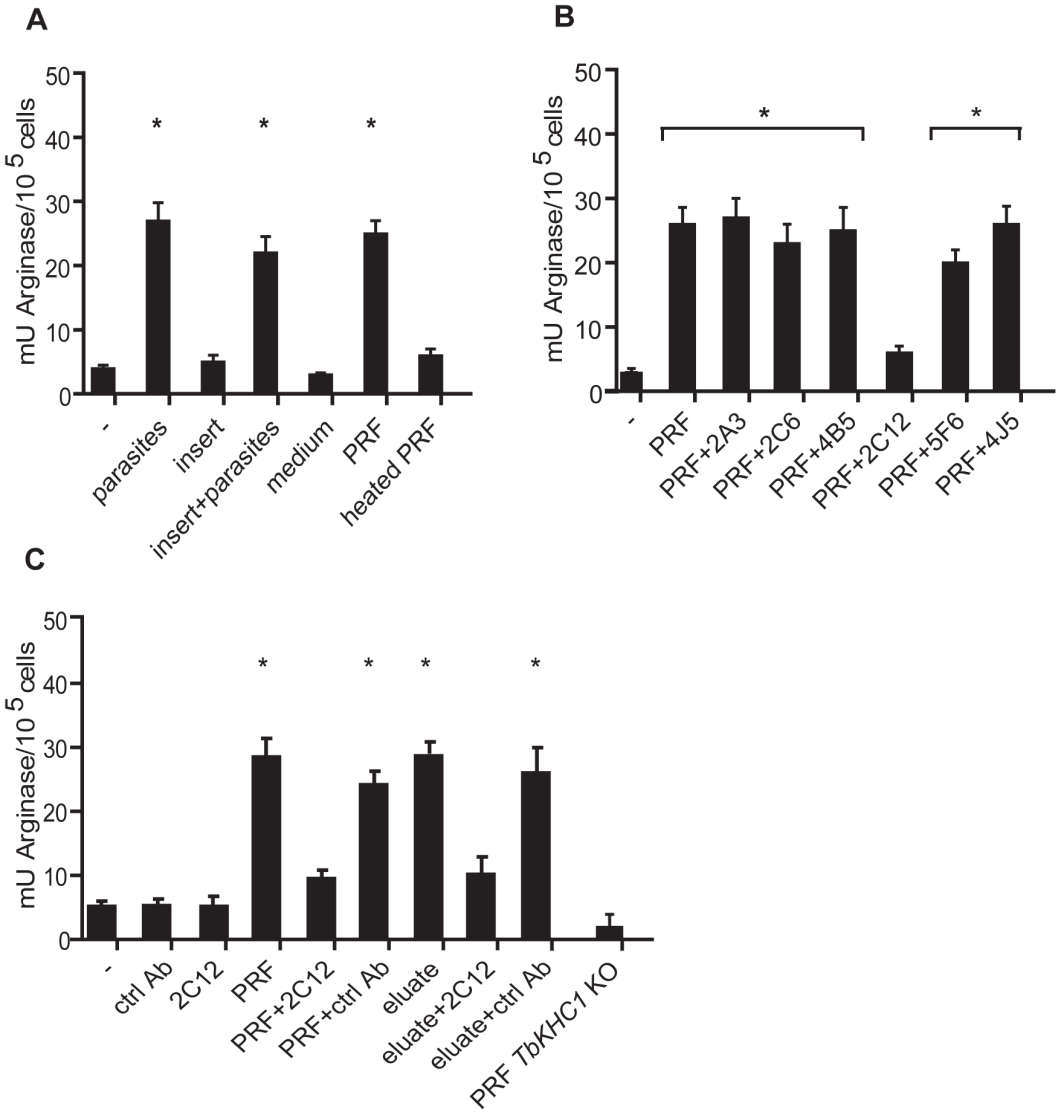
Induction of myeloid cell arginase activity by *T. brucei* PRF. Arginase activity was determined in myeloid cells from non-infected mice incubated with (**A**) WT parasites separated or not by an insert, or WT parasite-released factors (PRF), (**B**) PRF from WT parasites in presence of indicated antibodies and (**C**) PRF from WT or *TbKHC1* KO parasites, or material purified on anti-PRF antibody affinity column, in presence of indicated antibodies. Heat treatment was at 45°C for 30 min. Data are means ± SEM of 3–4 individual mice of one representative from 3 independent experiments (A, B), or of triplicate cultures of one representative from 2 independent experiments (C). * p<0.05 compared to non-stimulated (-) cells.

*T. brucei* parasites were found to induce arginase activity in myeloid cells from non-infected mice (Fig. 1A). This induction was maintained when myeloid cells and trypanosomes were separated by a cell-retaining insert, indicating that soluble components from trypanosomes were involved (Fig. 1A). Parasite-released factors (PRF) were prepared under conditions leading to no detectable trypanosome death. PRF induced arginase activity, and this effect was abolished by heat-treatment (Fig. 1A). Monoclonal antibodies raised against *T. brucei* PRF (2C12) inhibited arginase activity induced by PRF, whereas other antibodies from the same hybridoma fusion (2A3, 2C6, 4B5) or of irrelevant specificity (5F6, 4J5) had no effect (Fig. 1B). The PRF fraction eluted after binding to a 2C12 antibody-based affinity column retained full arginase-inducing activity, confirming that this activity was directly targeted by 2C12 (Fig. 1C).

The 2C12 antibody was used to screen a cDNA expression library and identify the *T. brucei* arginase-inducing protein. Among the 6 positive clones identified, 5 encoded fragments of the same putative kinesin heavy chain isoform (geneDB accession number Tb927.6.4390), which we termed TbKHC1 for *T. brucei* kinesin heavy chain 1 (Fig. S1in Text S1). The protein N-terminal domain contained the highly conserved kinesin motor domain with a typical ATP binding site. The region targeted by 2C12 antibody located downstream from the motor domain and was characterized by a predicted coiled structure (Fig. S1 in Text S1).

*TbKHC1* knock-out (KO) parasites were generated from wild-type (WT) *T. brucei*. In contrast to PRF from WT parasites, PRF from *TbKHC1* KO parasites did not trigger arginase activity in myeloid cells from non-infected mice (Fig. 1C).

### TbKHC1 partially localizes in the endocytic compartment and is released from the parasite

**Figure 2:**
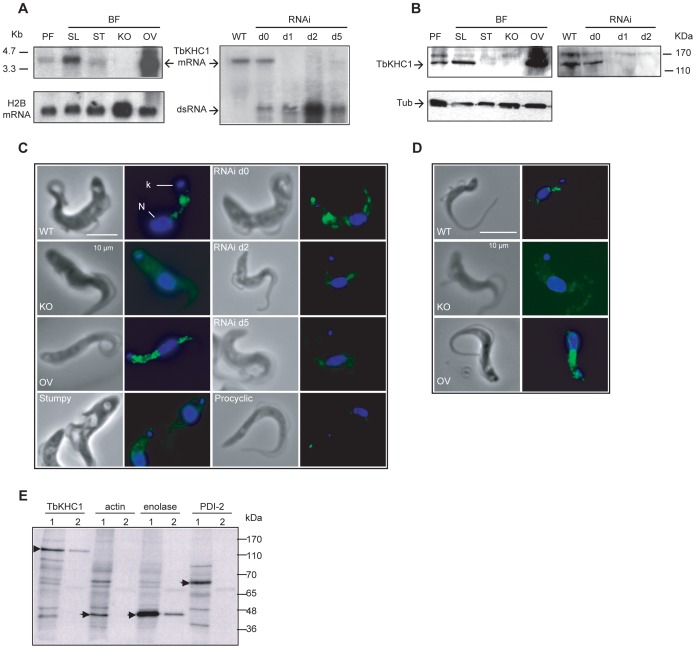
Expression and localization of *T. brucei* TbKHC1. (**A**) Detection of *TbKHC1* transcripts by Northern blotting of RNA from procyclic forms (PF), bloodstream (BF) slender (SL) and stumpy (ST) forms, BF *TbKHC1* knock-out (KO), overexpressor (OV) and *TbKHC1* knock-down (RNAi) trypanosomes (d: days after doxycyclin induction; ds RNA: double-stranded RNA; histone H2B mRNA: loading control). (**B**) Detection of TbKHC1 with anti-rTbKHC1 polyclonal antibody in whole extracts from the indicated trypanosome forms (Tub: Tubulin loading control). (**C**) Parasite staining with anti-PRF monoclonal 2C12 antibody. Nucleus (N) and kinetoplast (K) are stained in blue with 4′,6-Diamidino-2-phenylindole (DAPI). (**D**) Localization of TbKHC1 on WT, KO and OV trypanosomes with anti-rTbKHC1 polyclonal antibody. (**E**) Immunoprecipitation of TbKHC1, actin, enolase and PDI-2 from ^35^S-metabolically labelled WT parasite total extracts (1) or supernatants (2).

Pleomorphic trypanosomes, differentiating in the bloodstream from proliferative slender forms into quiescent stumpy forms, can induce long-standing infection in mammals and perform cyclical transmission in tsetse flies while differentiating into procyclic forms. In contrast, monomorphic trypanosomes, resulting from prolonged cultivation *in vitro*, are unable to develop long-lasting infection and cyclical transmission. The *TbKHC1* gene was found to be expressed preferentially in slender bloodstream forms (Fig. 2A,B). Parasite immunolabeling with both monoclonal 2C12 and polyclonal antibodies generated against recombinant TbKHC1 (rTbKHC1) vanished in *TbKHC1* KO parasites or RNAi-mediated *TbKHC1* knock-down (KD) parasites, while it increased in trypanosomes over-expressing TbKHC1 (Fig. 2A–D), confirming that TbKHC1 is the genuine target of the anti-PRF 2C12 antibody.

Both antibodies revealed a scattered protein distribution primarily between the kinetoplast and the nucleus (Fig. 2C,D). TbKHC1 partially co-localized with tomato lectin, which detects linear chains of poly-N-acetyllactosamine typical of the endocytic compartment [16] (Fig. S2A in Text S1). However, TbKHC1 did not bind to tomato lectin in affinity chromatography assay (Fig. S2B in Text S1), suggesting that TbKHC1 is not an endocytic component. Moreover, a fraction of TbKHC1 was reproducibly found in the culture medium of parasites, as revealed by immunoprecipitation of detergent-free supernatants from metabolically ^35^S-labelled *T. brucei* (Fig. 2E). In these experiments, trypanosome lysis was unlikely as the supernatants did not contain any evidence of the cytoplasmic markers actin [17] or protein disulfide isomerase 2 [18], but did contain enolase, a protein released from trypanosome-related parasites such as *Leishmania* [19] (Fig. 2E). TbKHC1 immunoprecipitated from the trypanosome supernatant did not associate with other ^35^S-labelled proteins (Fig. 2E), as could have been expected in case of release as a complex.

### IL-10 and mannose regulate TbKHC1-induced arginase activity

**Figure 3:**
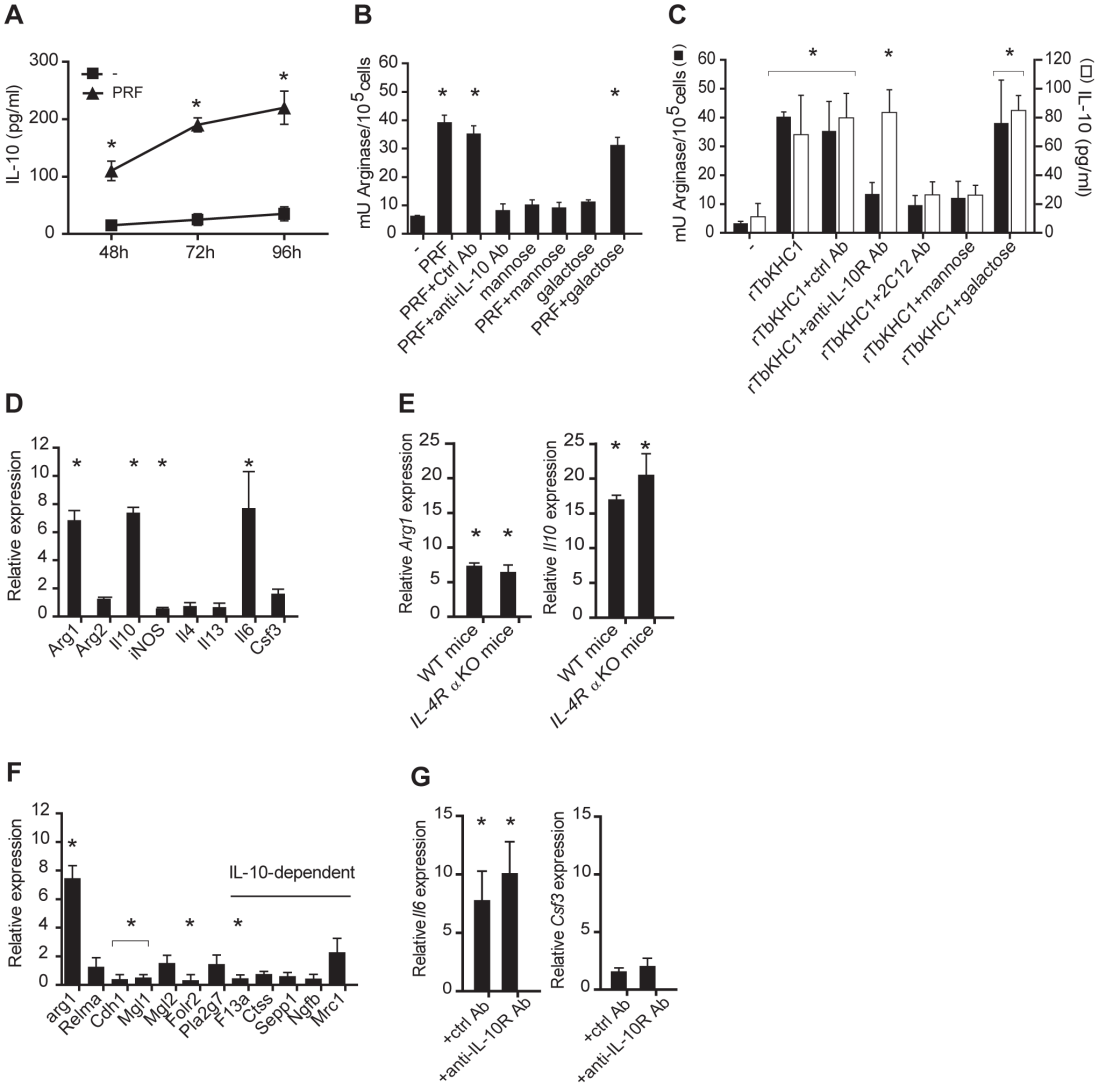
Mechanism of arginase activity induction by *T. brucei* PRF and rTbKHC1. Myeloid cells from non-infected WT (**A-D,F, G**) or IL-4Rα KO (**E**) mice were incubated with PRF or rTbKHC1. (**A**) IL-10 production induced by PRF. (**B**) Arginase activity induced by PRF incubated with indicated antibodies or sugars. (**C**) Arginase activity and IL-10 secretion induced by rTbKHC1 incubated with indicated antibodies or sugars. (**D–G**) Relative expression level of M2 genes after incubation with rTbKHC1. In **F**, M2 IL-10-dependent genes are indicated, and in **G**, rTbKHC1 was incubated with the indicated antibodies. Data are means ± SEM of 3 individual mice of one representative from 3 independent experiments. * p<0.05 compared to non-stimulated (-) cells.

We screened for pathways altering the levels of PRF-induced arginase activity in myeloid cells. Co-incident with the induction of arginase activity, PRF induced myeloid cells to express the regulatory cytokine IL-10 (Fig. 3A). The arginase activity induced by PRF was inhibited by neutralizing anti-IL-10 antibody and by D-mannose, but not by D-galactose (Fig. 3B).

rTbKHC1 mimicked the arginase-inducing effect of PRF, including its inhibition by 2C12, anti-IL-10R antibodies and D-mannose (Fig. 3C). The increased arginase activity induced by rTbKHC1 associated with increased expression of the *Arg1*, but not *Arg2* gene, as well as impaired expression of the *iNOS* gene (Fig. 3D). Activation of myeloid cells by rTbKHC1 also resulted in increased expression of the *IL-10* gene (Fig. 3D) and protein (Fig. 3C), the latter being inhibited by the 2C12 antibody and D-mannose. Thus, TbKHC1 interaction with myeloid cells involved a mannose-sensitive event triggering the synthesis of IL-10, resulting in enhanced *Arg1* gene expression and activity, and inhibition of *iNOS* expression.

Although arginase-1 expression is commonly associated with alternatively activated myeloid cell (M2) functions, rTbKHC1 did not trigger *Il4* and *Il13* gene expression in myeloid cells from WT mice (Fig. 3D). Moreover, the induction of *Arg1* and *Il10* genes by rTbKHC1 was conserved in *IL-4Rα* KO mice (Fig. 3E), excluding that IL-10 synergistically enhanced IL-4Rα-induced *Arg1* expression as observed in M2 cells [20], [21]. Accordingly, rTbKHC1 did not trigger the expression of M2-associated genes characteristic of African trypanosome infection [22] (Fig. 3F). We also addressed whether the induction of *Arg1* by rTbKHC1 required IL-6 and G-CSF in combination with IL-10 [23]. rTbKHC1 only induced *Il6* gene expression (Fig. 3D). This expression was not affected following addition of anti-IL-10R antibody that inhibited the induction of arginase activity by rTbKHC1 (Fig. 3G), indicating that IL-10 induced by TbKHC1 is the main trigger of *Arg1* expression in myeloid cells.

### TbKHC1 promotes parasite growth in mice by increasing ornithine availability

**Figure 4:**
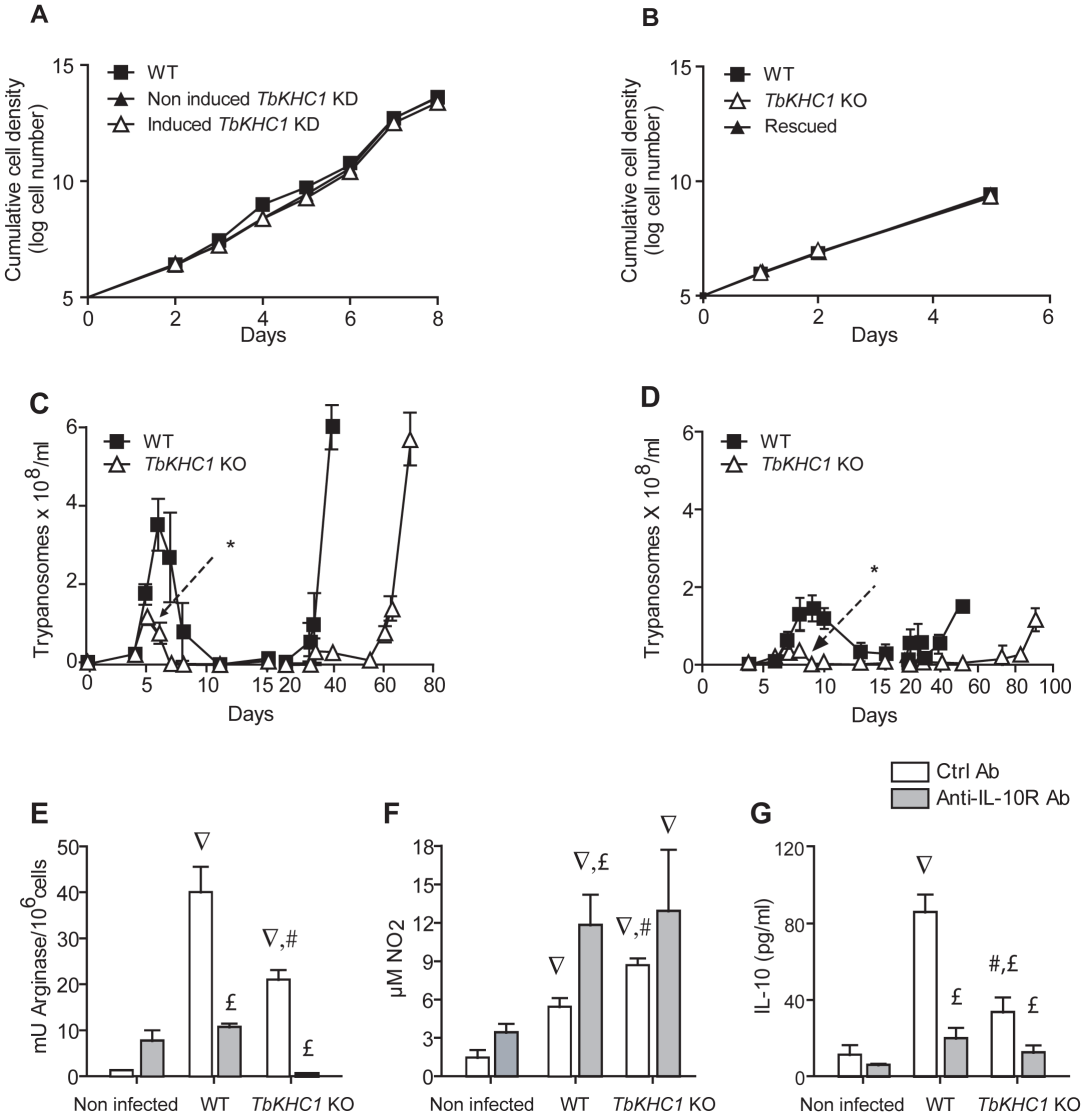
Effects of TbKHC1 on *T. brucei* growth and infection in mice. (**A,B**) *In vitro* parasite growth (**A**: monomorphic trypanosomes, either WT or induced and non-induced *TbKHC1* KD; **B**: pleomorphic trypanosomes, either WT or *TbKHC1* KO and rescued *TbKHC1* KO). Data are means ± SEM of one representative from 3 independent experiments (**C,D**) Parasitemia in C57Bl/6 mice infected with pleomorphic WT and *TbKHC1* KO parasites (**C**: intraperitoneal injection; **D**: injection through the bite of infected tsetse flies). Data are means ± SEM of 4 individual mice of one representative from 6 independent experiments. * p<0.05 comparing *TbKHC1* KO- and WT-infected mice. (**E–G**) TbKHC1-mediated myeloid cell activation. At day 6 p.i. with WT and *TbKHC1* KO parasites, mice were treated with anti-IL-10R (anti-IL-10R Ab) or control antibody (Ctrl Ab), and at day 7 spleen myeloid cells were analyzed for arginase activity (**E**), spontaneous NO secretion (**F**) and IL-10 secretion (**G**). Data are means ± SEM of 3 individual mice of one representative from 3 independent experiments. ∇ p<0.05 comparing WT- or *TbKHC1* KO-infected to non-infected mice; £ p<0.05 comparing WT- or *TbKHC1* KO-infected mice treated with anti-IL-10R antibody to WT- or *TbKHC1* KO-infected control antibody-treated mice; # p<0.05 comparing *TbKHC1* KO- and WT-infected mice treated with control antibody.

*In vitro*, the parasite growth rate appeared to be identical between WT, *TbKHC1* KD, *TbKHC1* KO and *TbKHC1*-rescued parasites obtained by reinsertion of the *TbKHC1* gene in one allele of its original locus in the *TbKHC1* KO genome (Fig. 4A,B), indicating that TbKHC1 is not required for parasite growth. Moreover, *TbKHC1* KO parasites motility or flagellum movement addressed by microscopic examination and sedimentation analysis [24] appeared normal (not shown). *TbKHC1* KO parasites were able to perform the full parasite life-cycle, since procyclic transformants could cycle through tsetse flies to produce infective metacyclic forms.

In C57Bl/6 mice, the cumulative parasite load in the first peak of parasitaemia by *TbKHC1* KO trypanosomes was reduced by >70% as compared to WT parasites (Fig. 4C). This reduced *TbKHC1* KO parasite load was also observed under natural infection conditions in which infected tsetse flies were allowed to feed on mice (Fig. 4D). Reinsertion of *TbKHC1* in *TbKHC1* KO parasites reverted early parasitaemia to WT levels (Fig. S3 in Text S1).

As compared to infection with WT parasites, at day 7 post-infection (p.i.) with *TbKHC1* KO parasites, spleen myeloid cells showed a reduced induction of arginase activity that coincided with increased NO_2_ accumulation, likely reflecting a shift towards NOS activity (Fig. 4E,F). Moreover, the induction of arginase activity was not observed in infected mice treated with a neutralizing anti-IL-10R antibody (Fig. 4E). Furthermore, mice infected with *TbKHC1* KO parasites exhibited lower IL-10 serum levels than mice infected with WT parasites (Fig. 4G). The reduction of IL-10 secretion in *TbKHC1* KO-infected mice could account for the higher inhibition of arginase activity observed in the presence of anti-IL-10R antibodies in these mice (Fig. 4E).

**Figure 5:**
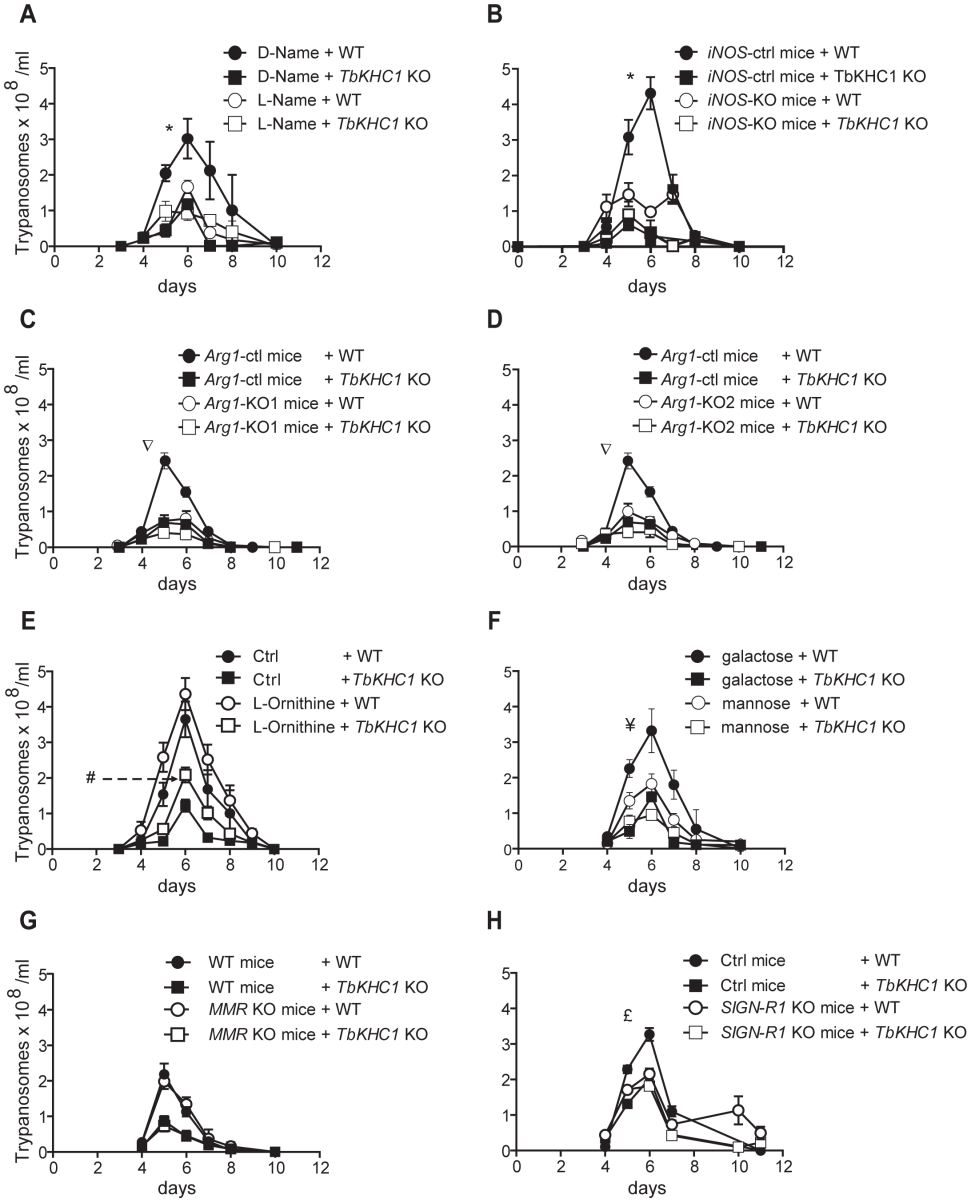
Effects of TbKHC1 on *T. brucei* parasitaemia. WT and *TbKHC1* KO parasitaemias were monitored in various mice and conditions: (**A**) WT mice treated with L-NAME or D-NAME; (**B**) *iNOS* KO and WT mice; (**C,D**) myeloid cell *Arg1* KO mice (KO1 = *LysM* ^cre^ *Arg1* ^-/lox^; KO2 = *Tie2* ^cre^ *Arg1* ^-/lox^) and controls (*Arg1* ^lox/lox^); (**E,F**) WT mice treated with L-ornithine, D-mannose or D-galactose; (**G**) *MMR* KO and WT mice; (**H**) *SIGN-R1* KO and control (Ctrl) mice. Data are means ± SEM of 4 individual mice of one representative from 3 independent experiments. * p<0.05 comparing D-NAME and L-NAME treated mice (A) or WT and *iNOS* KO mice (B) infected with WT parasites; ∇ p<0.05 comparing *Arg1* ^lox/lox^ and *LysM* ^cre^ *Arg1* ^-/lox^ or *Tie2* ^cre^ *Arg1* ^-/lox^ mice infected with WT parasites; # p<0.05 comparing L-ornithine treated and non treated mice infected with *TbKHC1* KO parasites; ¥ p<0.05 comparing D-mannose and D-galactose-treated mice infected with WT parasites; £ p<0.05 comparing *SIGN-R1* KO and control mice infected with WT parasites.

In *TbKHC1* KO-infected mice, it is unlikely that the reduction of the first peak of parasitaemia resulted from increased NO production. Indeed, inhibition of NOS activity by N-(G)-nitro-L-arginine methyl ester (L-NAME) or absence of *iNOS* activity in *iNOS* KO mice did not affect *TbKHC1* KO parasitaemia, while it strongly reduced WT parasitaemia (Fig. 5A,B). The lower parasitaemia in *TbKHC1* KO-infected mice might rather result from reduced arginase activity, which would limit nutrient availability for the parasite. Indeed, this enzyme converts L-arginine to L-ornithine, a precursor of polyamines that are required for trypanosome growth [13]. Accordingly, in mice lacking arginase-1 in myeloid cells/macrophages following a cross between *Arg1* loxP-targeted mice and *LysM* ^Cre^ or *Tie2* ^Cre^ deleter mice [21], WT parasitaemia dropped to that of *TbKHC1* KO parasitaemia (Fig. 5C,D). Moreover, treatment of mice with L-ornithine increased the cumulative parasite load to a greater extent in *TbKHC1* KO- than in WT-infected mice (∼94% vs ∼43%, respectively; Fig. 5E). In addition, spermine levels tended to increase although without reaching statistical significance in spleen myeloid cells and blood from mice infected with WT but not *TbKHC1* KO parasites (Fig. S4A in Text S1). Furthermore, an increase in L-ornithine production, coinciding with the consumption of L-arginine and the induction of N-acetylputrescine production, was observed in the supernatant of myeloid cells from non-infected mice activated in vitro with rTbKHC1 (Fig. S4B in Text S1).

### The effect of TbKHC1 on parasite growth involves the myeloid cell receptor SIGN-R1

**Figure 6:**
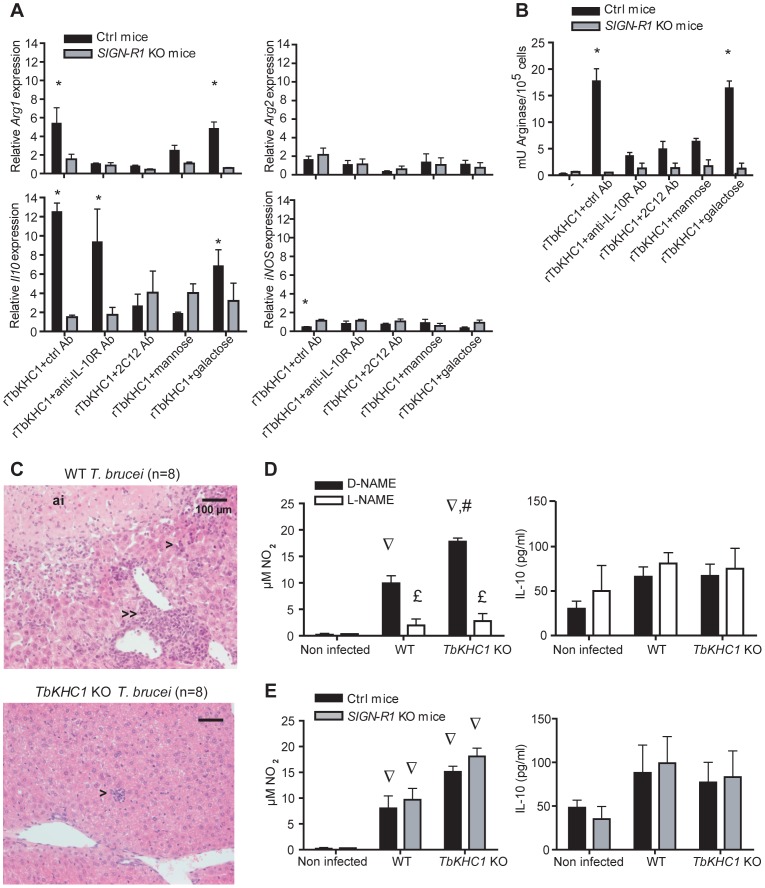
SIGN-R1 receptor contribution to myeloid cell activation and liver injury. (**A,B**) Effects of rTbKHC1 on myeloid cells from non-infected control (Ctrl) or *SIGN-R1* KO mice (**A**: relative expression of *Arg1*, *Arg2*, *Il10* and *iNOS* genes; **B**: arginase activity in presence of indicated antibodies or sugars). Data are means ± SEM of 3 individual mice of one representative from 3 independent experiments. * p<0.05 compared to non-stimulated (-) cells. (**C**) Effects of rTbKHC1 on liver injury: microscopic analysis (hematoxylin-eosin staining, magnification 20×) of sections from WT- and *TbKHC1* KO-infected mice at day 30 p.i. Anoxic infarcts (ai), periportal (>>) and lobular (>) mononuclear cell infiltrates were representative of 8 animals tested in 2 independent experiments. (**D**) Spontaneous NO and IL-10 secretions in spleen myeloid cell supernatants from WT- and *TbKHC1* KO-infected mice treated with D-NAME or L-NAME (day 30 p.i.). (**E**) Idem as **D** in *SIGN-R1* KO and control (Ctrl) mice. Data are means ± SEM of 3–4 individual mice of one representative from 3 independent experiments. ∇ p<0.05 comparing WT or *TbKHC1* KO- infected mice to non-infected mice; £ p<0.05 comparing L-NAME and D-NAME treatment in WT- or *TbKHC1* KO-infected mice; # p<0.05 comparing WT- and *TbKHC1* KO-infected mice.

Since the *in vitro* induction of arginase activity by PRF and rTbKHC1 was inhibited by D-mannose (Fig. 3B,C), we treated infected mice with D-mannose. This treatment reduced WT parasitaemia to the level of *TbKHC1* KO parasitaemia, but did not significantly affect *TbKHC1* KO parasitaemia (Fig. 5F). To follow up on this *in vivo* finding and the *in vitro* observation that arginase activation by PRF and rTbKHC1 was inhibited by D-mannose as well as by an antibody recognizing coils of TbKHC1 (Fig. 1B, 3C), we monitored the course of WT- and *TbKHC1* KO-infection in mice deficient for myeloid cell receptors able to bind both mannose and peptidic coils, namely MMR (CD206) and SIGN-R1 (CD209b), using appropriate congenic control mice [25]–[27]. In *MMR* KO mice, WT and *TbKHC1* KO parasitaemias were not affected as compared to infection in WT mice (Fig. 5G). In *SIGN-R1* KO animals the differential control of the first peak of parasitaemia between WT and *TbKHC1* KO parasites vanished, due to the reduction of the WT parasite level to that of *TbKHC1* KO parasites (Fig. 5H). Thus, either the absence of SIGN-R1 in infected mice or the absence of TbKHC1 in the parasite decreased *T. brucei* parasitaemia similarly. These data suggested that TbKHC1 interacts with the SIGN-R1 receptor. To evaluate this hypothesis, we tested the *in vitro* activity of rTbKHC1 on myeloid cells from *SIGN-R1* KO mice. In contrast to results obtained with myeloid cells from control mice, in myeloid cells from *SIGN-RI* KO mice rTbKHC1 did not stimulate *IL-10* and *Arg1* gene expression (Fig. 6A) and did not increase arginase activity (Fig. 6B). Conversely, in myeloid cells from *MMR* KO mice rTbKHC1 induced expression of *Il10* and *Arg1* genes as in WT mouse myeloid cells (Fig. S5 in Text S1). Therefore, TbKHC1 appeared to trigger arginase activity through SIGN-R1-mediated signaling.

### TbKHC1 increases liver pathogenicity and reduces host survival by NO-dependent mechanisms

The survival time of *TbKHC1* KO-infected mice was significantly prolonged as compared to WT-infected animals (Fig. S6 in Text S1, Table 1). Death of mice infected with African trypanosomes is related to systemic inflammatory response syndrome (SIRS) that causes multiple organ failure [28]. Liver myeloid cells are critical to the clearance of trypanosomes from the bloodstream [29], hence maintaining liver integrity is fundamental to the host's ability to clear trypanosomes. At day 30 p.i. several indicators of liver damage differed between WT- and *TbKHC1* KO-infected mice: (i) the serum levels of the liver damage marker alanine aminotransferase (ALT) were lower in *TbKHC1* KO- than in WT-infected mice; (ii) anoxic infarcts were observed in none of the *TbKHC1* KO-infected mice, but in 6 out of 8 WT-infected mice; (iii) lobular and portal mononuclear cell infiltrates were milder in all *TbKHC1* KO- than in WT-infected mice (Table 1, Fig. 6C). Concomitantly, NO production was higher in *TbKHC1* KO- than in WT-infected mice, while IL-10 production was not induced and similar in all infected experimental groups (Fig. 6D). A moderate increase in *iNOS* mRNA expression level was also observed in myeloid cells from mice infected with *TbKHC1* KO parasites (4.2±1.5 vs 1.32±0.33 fold in mice infected with WT parasites, n = 3; p = 0.08). These data suggested that increased NO production protected liver integrity in *TbKHC1* KO-infected mice. Accordingly, L-NAME treatment or knock-out of *iNOS* activity in *TbKHC1* KO-infected mice reduced the survival time and increased ALT levels to those found during WT parasite infection (Table 1). L-NAME treatment or absence of *iNOS* activity in WT-infected mice did not affect the survival time, although it reduced the serum levels of ALT (Table 1). Therefore, a threshold of NOS activity, achieved in *TbKHC1* KO- but not in WT-infected mice, might be required during *T. brucei* infection to protect liver integrity, thereby contributing to extended host survival. This hepatoprotective effect of NO was not due to difference in parasite load in mice infected with WT and *TbKHC1* KO parasites (Fig. S7 in Text S1).

**Table 1:** Survival and liver pathology in *T. brucei* WT- and *TbKHC1* KO- infected mice.

Mice	Survival time (days, Mean ± SEM)		# animals tested	ALT (day 30 p.i.) (U/ml, Mean ± SEM)		# animals tested
	WT	*TbKHC1* KO		WT	*TbKHC1* KO	
D-Name treated	42±6.1	79±10*	11	753±95	80±69*	12
L-Name treated	45±5.1	46±5.6£	15	344±120£	216±51£	12
*iNOS* control	42±8.0	83±15*	6	618±124	110±69*	6
*iNOS* KO	38±14	42±6.1£	6	494±99	545±53£	6
Untreated	42±9.0	66±12*	24	783±65	94±79*	15
L-Ornithine treated	40±6.9	46±3.9£	12	649±123	325±45£	12
*Arg1* control^a^	35±4.8	74±10*	12	808±316	195±177*	4
*LysM* ^cre^ *Arg1* KO^b^	37±4.4	71±13*	7	852±142	270±146*	4
*Tie2* ^cre^ *Arg1* KO^c^	39±7.6	76±9.5*	8	1130±295	160±80*	4
*MMR* control	37±2.3	69±18*	6	555±234	193±173	6
*MMR* KO	43±11	59±11	6	558±111	145±114*	6
*SIGN-R1* control	38±10	81±18*	9	1010±300	270±10*	6
*SIGN-R1* KO	29±6.0	95±12	13	745±187	360±50£	6

a *Arg1* ^lox/lox^ mice;

b *LysM* ^cre^ *Arg1* ^-/lox^ mice;

c *Tie2* ^cre^ *Arg1* ^-/lox^ mice.

* p<0.05 comparing *TbKHC1* KO- and WT- infected mice ;

£ p<0.05 comparing treated and control treated infected mice.

The survival times and ALT levels in WT and *TbKHC1* KO-infected mice were similar in *Arg1*-deficient mice and control mice (Table 1). However, treatment of WT-infected mice with L-ornithine slightly increased the serum level of ALT, while in *TbKHC1* KO-infected mice L-ornithine treatment reduced the survival time and increased ALT levels to those found during WT parasite infection (Table 1). Therefore, L-ornithine as a polyamine precursor favored the development of liver injury and negatively affected the survival of *T. brucei*-infected mice.

As in the liver, brain injury characteristic of African trypanosomiasis was also lower in *TbKHC1* KO- than in WT-infected mice. Indeed, no parasites could be detected in the choroid plexus of *TbKHC1* KO-infected mice, sharply contrasting with the presence of numerous parasites, associated with immune infiltrates, in the choroid plexus of WT-infected mice (Fig. S8 in Text S1).

The survival times as well as ALT, NO and IL-10 levels in WT- and *TbKHC1* KO-infected mice were similar in *SIGN-R1* KO and control mice (Table 1, Fig. 6E), suggesting that in late infection TbKHC1 signaling no longer operates primarily through SIGN-R1.

## Discussion

We report that a particular isoform of the large KHC family of *T. brucei* is released by the trypanosome and influences both parasite growth and host pathogenicity.

*T. brucei* contains 51 sequences encoding kinesin-like proteins. The *T. brucei* isoform described here appears to be an orphan member without predicted function [30]. That a KHC isoform could be released in the extracellular environment is not unprecedented, since a KHC-like protein has been reported as secreted from the coagulating gland in rat [31]. TbKHC1 did not appear to be contained in high molecular weight complexes released from the parasite, for instance in exosomes as occurs in *T. cruzi* [32] and *Leishmania* [33]. Our immunoprecipitation data, rather, suggested that TbKHC1 is free in the medium, but the mechanism of release remains unclear.

TbKHC1 did not appear to be essential for *T. brucei* cellular proliferation or differentiation.

*In vitro*, TbKHC1 triggered arginase activity and, in contrast to the other documented *T. brucei* immunomodulators VSG, DNA or TSIF [9], [10], [12], inhibited NO synthesis by myeloid cells. These effects involved a region rich in coils in the TbKHC1 C-terminal domain, since they were inhibited by an antibody specifically recognizing the coiled region of TbKHC1.

*Arg1* is often recognized as a prototypic M2 myeloid cell marker induced following STAT6 activation by IL-4/IL-13/IL-4Rα signaling. However, *Arg1* can be expressed by M1 cells in a STAT3-dependent pathway triggered by IL-10, IL-6 and G-CSF [21], [23]. In agreement, rTbKHC1 induced the expression of *Il10* and *Il6* genes in myeloid cells, and this neither involved IL-4Rα nor induced the expression of genes associated with M2 activation. Since *Il6* expression was still induced in the presence of the *Arg1* expression inhibitor anti-IL-10R antibody, we conclude that rTbKHC1-induced *Arg1* expression primarily depended on IL-10 production.

In mice, TbKHC1 activity appeared to promote parasite growth in the early stage of infection. Reduced parasitaemia induced by *TbKHC1* KO trypanosomes was associated with increased NO production, but did not directly result from this increase. Indeed, inhibition of NO production by L-NAME treatment or in *iNOS* KO mice did not improve *TbKHC1* KO growth. Rather, in agreement with previous studies [34] L-NAME treatment or absence of *iNOS* activity in mice actually increased the control of WT parasitaemia. Together, these experiments suggested that the negative/braking effect of NO on the control of WT parasitaemia requires the expression/activity of TbKHC1. We propose that TbKHC1-mediated induction of host arginase activity fuels L-ornithine production and hereby the synthesis of polyamines, which are essential nutrients for growth of extracellular trypanosomes in the host. In bloodstream forms of *T. brucei* the arginase gene and activity are absent, but when exogenous L-ornithine is lacking in body niches these parasites can use L-arginine to produce L-ornithine through an arginase-independent mechanism [35], [36]. However, the differential growth-promoting effect of exogenous L-ornithine during infection by WT or *TbKHC1* KO parasites suggests that this arginase-independent mechanism is insufficient to provide all the amines necessary for the polyamine pathway. Similarly, the related but intracellular parasite *Leishmania* also requires host arginase induction for optimal growth, despite the presence of an arginase gene [37]. Therefore, optimal production of L-ornithine/polyamines for *T. brucei* growth would rely on host arginase activity induced by TbKHC1. In accordance, in two models of mice deficient for arginase-1 in myeloid cells/macrophages, WT *T. brucei* growth was reduced to the level observed with *TbKHC1* KO parasites.

In *T. brucei*-infected mice, the TbKHC1-mediated effect on parasitaemia appeared to involve the myeloid cell receptor SIGN-R1. Indeed, improved parasite growth control was observed after either removal of TbKHC1 from the parasite or removal of SIGN-R1 from the host. Moreover, rTbKHC1-mediated induction of arginase activity was lost in myeloid cells from *SIGN-R1* KO mice. The involvement of SIGN-R1 in TbKHC1-mediated IL-10 and arginase production is in keeping with previous data demonstrating the anti-inflammatory role of SIGN-R1 in various diseases [38]–[40]. Although LPS and intracellular mycobacteria infection can induce *Arg1* in a MyD88/IL-10 dependent manner [21], [23], we can exclude that TbKHC1 is simultaneously a ligand for SIGN-R1 and a trigger for MyD88 signaling in our extracellular infection model since the differential infection phenotype between WT and *TbKHC1* KO *T. brucei* was conserved in *Myd88* KO mice (data not shown).

D-mannose was found to decrease parasite growth during the first peak of parasitaemia by WT but not *TbKHC1* KO parasites, and it inhibited rTbKHC1-mediated induction of IL-10 and arginase by myeloid cells. Similarly, the 2C12 antibody, which is specific to the coiled region of TbKHC1, inhibited IL-10 production and arginase activity triggered by rTbKHC1. Mannose-specific receptors such as SIGN-R1 and MMR are known to interact with coiled proteins, the complement protein C1q and collagen respectively [26], [27]. Therefore, it is possible that mannose binding to SIGN-R1 could affect the binding of TbKHC1 peptidic coils to a second binding site of this receptor.

After the parasite growth-promoting effect occurring during the first peak of parasitaemia, a distinct effect of TbKHC1 consisted in reducing the survival time of infected mice while contributing to liver injury. Given the similar *in vitro* growth properties between parasites expressing or not TbKHC1, the mechanism by which this protein influenced the host survival time is likely to be independent from differences in parasite virulence. Parasite load throughout the infection, survival and pathogenicity are independent traits in experimental and natural infection with African trypanosomes [2], [4], [6]–[8], [28]. Therefore, the survival time difference between WT- and *TbKHC1* KO-infected mice is primarily due to differences in tissue damage inflicted by inflammatory immune responses, rather than differences of parasite load in the first peak of parasitaemia. NO contributes to peripheral pathogenicity induced by African trypanosomes, while IL-10 exerts counter-pathogenic activity [2], [3]. In late stages of infection, IL-10 production was unaffected by the absence of TbKHC1, while NO production (and iNOS expression) was lower when TbKHC1 was present. Moreover, the reduction of liver injury in *TbKHC1* KO-infected mice was erased by inhibition of NO synthesis upon L-NAME treatment or by infecting *iNOS* KO mice. Thus, TbKHC1-mediated depletion of NO might contribute to the liver damage associated with WT parasite infection. Accordingly, NO plays a pivotal role in vascular tone, and decreased NO bioavailability contributes to pathogenesis in chronic infectious diseases like malaria, and in liver ischemia reperfusion injury [41], [42]. Our observation that after the first peak of parasitaemia parasite load was similar in WT- and *TbKHC1* KO -infected mice, even upon inhibition of NO production, supports the notion that blood parasite load is not a major contributor to liver pathogenicity.

Besides NO, polyamines contribute to the pathogenicity of *T. brucei* infection [43]. Since our data suggest that induction of arginase activity by TbKHC1 could sustain the production of polyamines, these molecules could contribute to increase liver injury in WT- as well as in *TbKHC1* KO-infected mice fed with L-ornithine.

In the late stage of infection, the effects of TbKHC1 did not appear to depend on SIGN-R1 any longer, since the absence of SIGN-R1 did not affect the survival time and liver injury in WT- and *TbKHC1* KO-infected mice. A major difference between early and late infection is the evolution of the immune response driven by the continuous release of immunomodulatory/inflammatory factors by the parasite, such as VSG and TSIF, which induce pathogenic TNF-α and IFN-γ [1], [4], [9] while switching off the IL-10 production [7]. The evolving context of host-parasite interaction could affect TbKHC1 signaling, and indeed the myeloid cell activation state and corresponding expression of receptors is known to vary during the time course of *T. brucei* infection [44]. Therefore, we cannot exclude that in infected *SIGN-R1* KO mice, compensation by other myeloid cell receptors influences the immune response and outcome of pathogenicity in the late stage of the disease.

In conclusion, we have identified an unexpected function for a KHC of extracellular *T. brucei*, which is SIGN-R1-mediated activation of myeloid cells to produce IL-10 leading to increase in arginase activity, resulting in promotion of parasite growth in the host.

## Methods

### Ethic statement

Experiments, maintenance and care of mice complied with guidelines of the European Convention for the Protection of Vertebrate Animals used for Experimental and other Scientific Purposes (CETS n°123) and were approved by the Ethical Committee for Animal Experiments of the Université Libre de Bruxelles, Brussels, Belgium (laboratory accreditation number LA2500482).

### Mice, trypanosomes and infection protocol

WT C57Bl/6 mice were from Harlan, *iNOS* KO C57Bl/6 mice from Jackson Laboratory. *MMR* KO, *IL4Rα* KO, control *Arg1* ^lox/lox^, *LysM* ^cre^ *Arg1* ^-/lox^ and *Tie2* ^cre^ *Arg1* ^-/lox^ mice (C57BL/6 background), *SIGN-R1* KO and congenic control mice (BALB/c background) were bred in-house.

*T. brucei* bloodstream monomorphic “single-marker” [45] and pleomorphic AnTat 1.1E (EATRO1125 strain) were used.

Mice were infected i.p. with 2,000 AnTat1.1E parasites [6]. Parasitaemia was monitored by tail blood puncture. When required, mice were injected i.p. 2 h before infection with 2 mg L-NAME, then 1 mg/ml L-NAME was added to drinking water. The less active enantiomer D-NAME was used as control. For L-ornithine treatment, mice received 2 mg i.p. 2 h before infection, and then were given 5 mg/ml in drinking water. For D-mannose or D-galactose treatment, mice received 50 mg i.p. 2 h before infection, then every 2 days. Sugar treatment caused massive inflammation and the animals were euthanized 12 days p.i. To neutralise IL-10 activity, mice were treated once with 200 µg control antibody (rat IgG1, BD Biosciences) or 1B1.3a anti-IL-10R antibody at day 6 p.i. Injections of anti-IL-10R antibody at day 2, 4 and 6 p.i. killed WT- and *TbKHC1* KO- infected mice within 48 h, as reported [6].

### Parasite released factors, antibodies and PRF immunoaffinity column

PRF was prepared as described [15]. Monoclonal antibodies were obtained following an immunoenzymatic screening that selected antibodies directed against PRF, followed by a test based on inhibition of arginase activity induction. rTbKHC1 C-terminal fragment was used for production of rabbit polyclonal antibodies. PRF was incubated overnight with 2C12 monoclonal antibody coupled to Sepharose 4B CNBr resin at 4°C in carbonate buffer (v/v). After loading on a column and washings, bound material was eluted with glycine-HCl (pH 3), then glycine-NaOH (pH 10), dialysed for 24 h against milliQ water, lyophilised and solubilized at 1 mg/ml in 50 mM phosphate buffer, 1 M NaCl (pH 6.2). The residual level of endotoxins was below 2 U/µg (LAL assay, Biowhittaker).

### cDNA expression library screening

Approximately 10^5^ plaques from a bloodstream *T. b. gambiense* LiTat1.3 cDNA expression library (lambda ZAP express, Stratagene) were screened by incubation with the mouse 2C12 monoclonal antibody. Clones targeted by an anti-mouse antibody/alkaline phosphatase conjugate were identified by incubation at pH 9.5 with the chromogenic substrate NBT/BCIP. Reactive clones were plaque purified by 2 additional rounds of screening. The positive clones were processed to obtain the circular pBK CMV plasmid version of the phage, according to the manufacturer instructions.

### *TbKHC1* KD and KO parasites

For *TbKHC1* KD, a 500 bp fragment from the *TbKHC1* open reading frame (ORF) was amplified by polymerase chain reaction (PCR) using the KD primers (Table S1 in Text S1), digested with *Xho*I and *Bam*HI and ligated into the p2T7-177TiTA plasmid [46]. The vector was linearized with *Not*I and transfected into monomorphic bloodstream forms. Stable transformants were selected with 1 µg/ml hygromycin. Double stranded RNA production was induced by addition of 1 µg/ml doxycyclin. For *TbKHC1* KO parasites, both alleles of the complete *TbKHC1* ORF were replaced in procyclic forms by genes encoding resistance to neomycin and bleomycin, following transfection of these genes flanked by 75 bp sequences from the *TbKHC1* UTRs. Primers used for PCR amplification of the resistance genes were KOneo and KObleo (Table S1 in Text S1). Stable transformants were selected with 15 µg/ml geneticin and 2.5 µg/ml phleomycin. Pleomorphic *TbKHC1* KO parasites were obtained by cyclical transmission of *TbKHC1* KO procyclic forms in tsetse flies.

### Rescue of *TbKHC1* KO parasites

*TbKHC1* gene and UTRs were PCR-amplified (Rescue primers, Table S1 in Text S1) and cloned into pCR-XL-TOPO vector (Invitrogen). The neomycin resistance cassette was extracted from pLew114hyg5′ [45] with *Afl*II and *Stu*I restriction enzymes and cloned into the *Afl*II and *Eco*RV digestion of the pCR-XL-TOPO-TbKHC1 clones presenting the 5′ sequence of TbKHC1 gene next to the pUC origin. pCR-XL-TOPO-TbKHC1-Neo was linearized at *Avr*II and transfected *in vivo* into *TbKHC1* KO pleomorphic parasites as described [47].

### TbKHC1 overexpressing trypanosomes

*TbKHC1* ORF was amplified by PCR from the initial pBK-CMV plasmid clone 4 (primers OV, Table S1 in Text S1) digested with *EcoR*V and *Hind*III, and cloned in the pTSARib vector [48]. The construct was linearized with *Bgl*II before transfection.

### rTbKHC1

The full length *TbKHC1* and a 2,235 bp *TbKHC1* fragment were amplified by PCR from the initial pBK-CMV plasmid clone 4 using primers FL and C-term (Table S1 in Text S1). The products were digested with *Sal*I and *Not*I and cloned in-frame in pET 21d (Novagen). rTbKHC1 was extracted from IPTG-induced *E. coli* and purified on Ni-NTA agarose (Qiagen) using 60% isopropanol in the washing steps to remove endotoxins [49]. The protein was additionally purified by HPLC Superdex 200 (10/300 GL, Pharmacia) in 50 mM phosphate buffer, 1 M NaCl (pH 6.2). The residual level of endotoxins was below 2 U/µg (LAL assay, Biowhittaker).

### Immunofluorescence assay

Fixed parasites (4% formaldehyde in PBS, 10 min, 20°C) spread on poly(L-lysine)-coated slides were permeabilized (0.1% Triton X-100 in Tris-buffered saline 5 min, 20°C) and incubated with 5% BSA. TbKHC1 was detected with the 2C12 monoclonal (dilution 1/200) or rabbit polyclonal (dilution 1/1000) antibodies and Alexa 488-conjugated anti-mouse or anti-rabbit IgG (dilution 1/1000).

### Metabolic labelling

Washed trypanosomes were incubated 15 min at 37°C at a density of 2×10^7^ cells/ml in IMDM methionine-free medium supplemented with 10% FCS, 0.07 mM L-arginine, 0.2 mM L-glutamine, 0.04 mM L-leucine, 0.1 µM inositol, 0.05 mM D-glucose, 30 mM Hepes, 1 mg/ml BSA, 0.5 mM adenosine and 5 µg/ml catalase. Then, a mix of ^35^S-labelled methionine and cysteine was added (100 µCi/ml). After 1 h labelling, cells were washed and incubated for 2 h at 37°C at a density of 2×10^6^/ml. Trypanosomes were centrifuged and lysed in 50 mM Tris, 150 mM NaCl, 2 mM EDTA, 1% NP40, complete protease inhibitors (Roche).

### Tomato lectin chromatography

Fractionation of trypanosome extracts on tomato lectin affinity column was performed as described [16].

### Preparation of myeloid cells

Non elicited peritoneal cells were collected in 0.34 M sucrose. Spleen homogenates were incubated 10 min in cold 0.83% NH_4_Cl/0.01 M Tris-HCl, pH 7.2 to lyse erythrocytes. Peritoneal and spleen cells were washed twice in complete medium (RPMI 1640 supplemented with 10% heat-inactivated FCS, 5×10^−5^ M 2-mercaptoethanol, 2 mM L-glutamine, 100 IU/ml penicillin, 100 µg/ml streptomycin, and 0.1 mM non essential amino acids; all from Invitrogen Life Technologies) and counted by trypan blue exclusion. To prepare myeloid cells, peritoneal cells adjusted to 5×10^6^ cells/ml in complete medium were dispensed by 1 ml in 6-well plates and spleen cells adjusted to 10^7^ cells/ml were dispensed by 10 ml in 10-cm tissue culture dishes. After 3 h at 37°C in 5% CO_2_, cells were washed five times with warm complete medium to remove non adherent cells. Adherent cells were collected in cold complete medium using a scraper, washed and viability was determined by trypan blue exclusion (usually >95%). Cells (CD11b^+^ for >90% as determined by flow cytometry, data not shown) were suspended in complete medium at 10^6^ cells/ml.

### In vitro culture of myeloid cells

Peritoneal myeloid cells from non infected mice (5×10^5^/ml) were cultured in 24-well plates in presence of 5×10^5^/ml trypanosomes, 80 µg/ml PRF, 3 µg/ml eluate of anti-PRF antibody affinity column or 3 µg/ml rTbKHC1. When required, antibodies (monoclonal anti-PRF, JES5-2A5 anti-IL-10 (BD Biosciences), 1B1.3a anti-IL-10R or appropriate isotype-matched antibodies (3 µg/ml), D-mannose or D-galactose (50 mM) were added to PRF- or rTbKHC1-stimulated cells. Unless specified, gene expression, arginase activity and NO_2_ and IL-10 secretions were determined after 24 h of culture. Alternatively, spleen myeloid cells from infected mice (10^6^/ml) were collected at day 7 or 30 p.i. and stored for arginase activity determination or cultured for 24 h before determining spontaneous NO_2_ and IL-10 production in culture supernatants.

### Myeloid cell metabolite extraction, HPLC-MS analysis and data processing

Spleen myeloid cells prepared as described above were cultured in modified complete medium (2% FCS, no 2-mercaptoethanol, no antibiotic) in 24-well plates in presence of 3 µg/ml rTbKHC1. After 12 h culture, supernatants were collected, rapidly cooled to 4°C in dry ice ethanol bath and centrifuged (3 min, 4°C, 13,000 g). Protein denaturation was achieved by adding 200 µl of 4°C chloroform∶methanol∶water (1∶3∶1) to 5 µl of cell supernatants. Samples were shaken for 1 h at 4°C and centrifuged again. The supernatants were extracted and frozen at −80°C until the samples were submitted to High Performance Liquid Chromatography-Mass Spectrometry (HPLC-MS) analysis at the Scottish Metabolomics Facility using High Performance Liquid Chromatography (Dionex UltiMate 3000 RSLC system) with a ZIC-HILIC 150×4.6 mm, 5 µm columns (Merck Sequant). The mobile phase comprised 0.1% formic acid in water (phase A) and 0.08% formic acid in acetonitrile (phase B). A linear gradient was applied to B from 80-20% over 30 minutes, followed by an 8 minute wash with 5% B and concluded with 8 minute equilibration with 80% B. The flow rate was 300 µL/min and 10 µL was injected into the column. The column temperature was 20°C and the autosampler temperature was 4°C.

For data processing, LC-MS raw data were processed using IDEOM software (version 17) [50] which is a composite of XCMS [51], mzMatch [52], R and Microsoft Excel packages. Firstly, XCMS detected peaks with a peak width between 10 and 100 seconds; signal to noise ratio of 5 and mass deviation between scans of 2 parts per million (ppm). MzMatch then matched the peaks between the two samples, comparing their retention times (RT) and ion densities before calculating any deviations. Peaks were identified according to their mass-to-charge ratio and RT.

### Arginase activity, NO_2_ and IL-10 secretion, spermine levels

Arginase activity was determined as described [53]. NO_2_ quantification (reflecting NO production) was assayed by a Griess reaction. IL-10 was quantified with a specific sandwich ELISA (PharMingen) in accordance to the manufacturers' protocols. Spermine levels (Spermine ELISA Kit, Antibodies-online) were determined in blood serum and myeloid cell homogenates following the manufacturers' protocols.

### Gene expression analysis

Quantitative real time PCR was performed as described [6]. Results of the PCR analyses were normalized against the house-keeping gene S12. Primers used are described in Table S2 in Text S1.

### Microscopy, ALT levels

Brains and livers were fixed in 4% formaldehyde. Histological sections embedded in paraffin were stained with hematoxylin-eosin-saffron for microscopic evaluations. ALT was measured in serum samples using commercially available kits (Boehringer Mannheim).

### Cumulative parasitaemia load, statistical analysis

GraphPad Prism 4.0 software was used to determine the cumulative parasitemia load (Area Under Curve function) and the statistical significance (Two-way ANOVA).

## Supporting Information

Text S1 Contains **Table S1** (Primers used to generate genetically modified parasites) and **Table S2** (Primers used for gene expression analysis in myeloid cells) as supplement to Experimental procedures. The **Text S1** contains also **Fig. S1** (Schematic primary structure of *T. brucei* TbKHC1), **Fig. S2** (Localization of *T. brucei* TbKHC1), **Fig. S3** (Characterization and growth pattern of *TbKHC1*-rescued *T. brucei*), **Fig. S4** (Effect of *T. brucei* infection on metabolites involved in L-arginine pathway), **Fig. S5** (Effect of rTbKHC1 on *Arg1* and *Il10* gene expression in *MMR* KO mice), **Fig. S6** (Effect of TbKHC1 on the survival of *T. brucei* infected mice), **Fig. S7** (Effects of absence of *iNOS* gene activity on *T. brucei* parasitaemia) and **Fig. S8** (Effects of TbKHC1 on cerebral injury in *T. brucei* infected mice), as well as their legends.
(DOCX)
