# Supplementary material for: A Trypanosoma brucei Kinesin Heavy Chain Promotes Parasite Growth by Triggering Host Arginase Activity
Source: PLoS Pathog. 2013 Oct 31;9(10):e1003731. doi: 10.1371/journal.ppat.1003731 (PMC3814429; doi:10.1371/journal.ppat.1003731)
Supplement: Text S1 — Contains Table S1 (Primers used to generate genetically modified parasites) and Table S2 (Primers used for gene expression analysis in myeloid cells) as supplement to Experimental procedures. The Text S1 contains also Fig. S1 (Schematic primary structure of T. brucei TbKHC1), Fig. S2 (Localization of T. brucei TbKHC1), Fig. S3 (Characterization and growth pattern of TbKHC1-rescued T. brucei), Fig. S4 (Effect of T. brucei infection on metabolites involved in L-arginine pathway), Fig. S5 (Effect of rTbKHC1 on Arg1 and Il10 gene expression in MMR KO mice), Fig. S6 (Effect of TbKHC1 on the survival of T. brucei infected mice), Fig. S7 (Effects of absence of iNOS gene activity on T. brucei parasitaemia) and Fig. S8 (Effects of TbKHC1 on cerebral injury in T. brucei infected mice), as well as their legends. (DOCX) [file ppat.1003731.s001.docx]

**Table S1: Primers used to generate genetically modified parasites ^a^**

| **Primers** | **Forward** | **Reverse** |
| --- | --- | --- |
| KD | 5’_CCGCTCGAGGCGGTGGCCGTCCCTTGCATAAC_3’ | 5’_CCCAAGCTTGGATCCTGCCTCTGGGTCGCTGGGGC_3’ |
| KOneo | 5’_TAGCTTGAGTTTTGCTTTATTTTTGTAGTTACTGTGCTTGTAGGGAATATACAATTTTGCTGCTGCTATGATTGAACAAGATGGATTGCACGC_3’ | 5’_GACATTGTATAAAAATCAGCAGTAAACAAAAAAGGAGAATAGCGGGGTGAGATTAAAACATGAGTCATAAAAAACTTGCATCAGAAGAACTCGTCAAGAAGGCG_3’ |
| KObleo | 5’_TAGCTTGAGTTTTGCTTTATTTTTGTAGTTACTGTGCTTGTAGGAATATACAATTTTGCTGCTGCTATGGCCAAGTTGACCAGTGCCGTTCCG_3’ | 5’_GACATTGTATAAAAATCAGCAGTAAACAAAAAAGGAGAATAGCGGGGTGAGATTAAAACATGAGTCATAAAAAACTTGCATCAGTCCTGCTCCTCGGCCACG_3’ |
| Rescue | 5’_ATCCACGGTGTGGGCTTGAGGTGTTAAGAAATAGACTCC_3’ | 5’_ATCTTAAGTCAGTTGGAGCGCAGGCGG_3’ |
| OV | 5’_CCCGATATCTCAGTTGGAGCGCAGGCGGC_3’ | 5’_TCAATTGGATCCAAGCTTATGTATCCGTATGACGTTCCAGACTACGCAGGTTTAAACATGTCGGATGCCGATGTGAAAGAGGG_3’ |
| GFP1 | 5’_GGGTCTAGATCGGATGCCGATGTGAAAGAGGG_3’ | 5’_GGGGGATCCCTCGAGGCGTGTGTTATGCAAGGGACG_3’ |
| GFP2 | 5’_GGGCTCGAGGCTTGAGGTGTTAAGAAATAGACTCC_3’ | 5’_GGGGGATCCAGCAGCAGCAAAATTGTATATTCCC_3’ |
| FL | 5’_ACGCGTCGACATGTCGGATGCCGATGTGAAAGAGGG_3’ | 5’_ATAAGAATGCGGCCGCGGGGTTGGAGCGCAGGCGGCG_3’ |
| Cterm | 5’_ACGCGTCGACTCATGCGAGCAATGGATGTGAAGGTGACG_3’ | 5’_ATAAGAATGCGGCCGCGGGGTTGGAGCGCAGGCGGCG_3’ |

^a^ Designed using the Primer-Blast software developed at NCBI, see Material and Methods for details

**Table S2: Primers used for gene expression analysis in myeloid cells ^a^**

| **Genes** | **Forward** | **Reverse** |
| --- | --- | --- |
| *Nos2* | 5’_TGGAGCCAAGGCCAAACACAG_3’ | 5’_TCCACCAGGAGATGTTGAAC_3’ |
| *Arg1* | 5'_AAGAAAAGGCCGATTCACCT_3' | 5'_CACCTCCTCTGCTGTCTTCC_3' |
| *Arg2* | 5'_ACAGGGTTGCTGTCAGCTCT_3' | 5'_TGATCCAGACAGCCATTTCA_3' |
| *Il10* | 5’_ACTCAATACACACTGCAGGTG_3’ | 5’_GGACTTTAAGGGTTACTTGG_3’ |
| *Il6* | 5’_GTCTTCTGGAGTACCATAGC_3’ | 5’_GTCAGATACCTGACAACAGG_3’ |
| *Csf3* | 5’_GGAGCTCTAAGCTTCTAGATC_3’ | 5’_TAGGGACTTCGTTCCTGTGAG_3’ |
| *Il4* | 5’_GTGCAGCTTATCGATGAATCC_3’ | 5’_AGCCATATCCACGGATGCGAC_3’ |
| *Il13* | 5’_GAAACAGTTGCTTTGTGTAGC_3’ | 5’_GGTGCCAAGATCTGTGTCTC_3’ |
| *Relma* | 5’_TCCCAGTGAATACTGATGAGA_3’ | 5’_CCACTCTGGATCTCCCAAGA_3’ |
| *Cdh1* | 5’_ACTTGGGGACAGCAACATCA_3’ | 5’_GGGTTTAAATCGGCCAGCAT_3’ |
| *Mgl1* | 5’_ATGATGTCTGCCAGAGAACC_3’ | 5’_ATCACAGATTTCAGCAACCTTA_3’ |
| *Mgl2* | 5’_GATAACTGGCATGGACATATG_3’ | 5’_TTTCTAATCACCATAACACATTC_3’ |
| *Folr2* | 5’_GGAGCTACACAAGGCTGAC_3’ | 5’_TGTGACAGGGTGCTGTGTTT_3’ |
| *Pla2g7* | 5’_TGCACCAGAACTTTGACGAC_3’ | 5’_CAACTGTGTAACAAGCAAGTTC_3’ |
| *F13a* | 5’_CCAGGAATTAAGCAAGACATC_3’ | 5’_TGCCCTTACTTCTAGTTCTC_3’ |
| *Ctss* | 5’_AGAAGGGCTGCGTCACTGAG_3’ | 5’_GAATGTACCTTGAACACGTAG_3’ |
| *Sepp1* | 5’_TTCTGCAGGCATCCAGATTG_3’ | 5’_CACAAGACGGCCACATCTGT_3’ |
| *Ngfb* | 5’_CATAGCGTAATGTCCATGTTGTTCT_3’ | 5’_CTTCTCATCTGTTGTCAACGC_3’ |
| *MMR* | 5’_GCAAATGGAGCCGTCTGTGC_3’ | 5’_CTCGTGGATCTCCGTGACAC_3’ |

^a^ Designed using the Primer-Blast software developed at NCBI, see Material and Methods for details

**
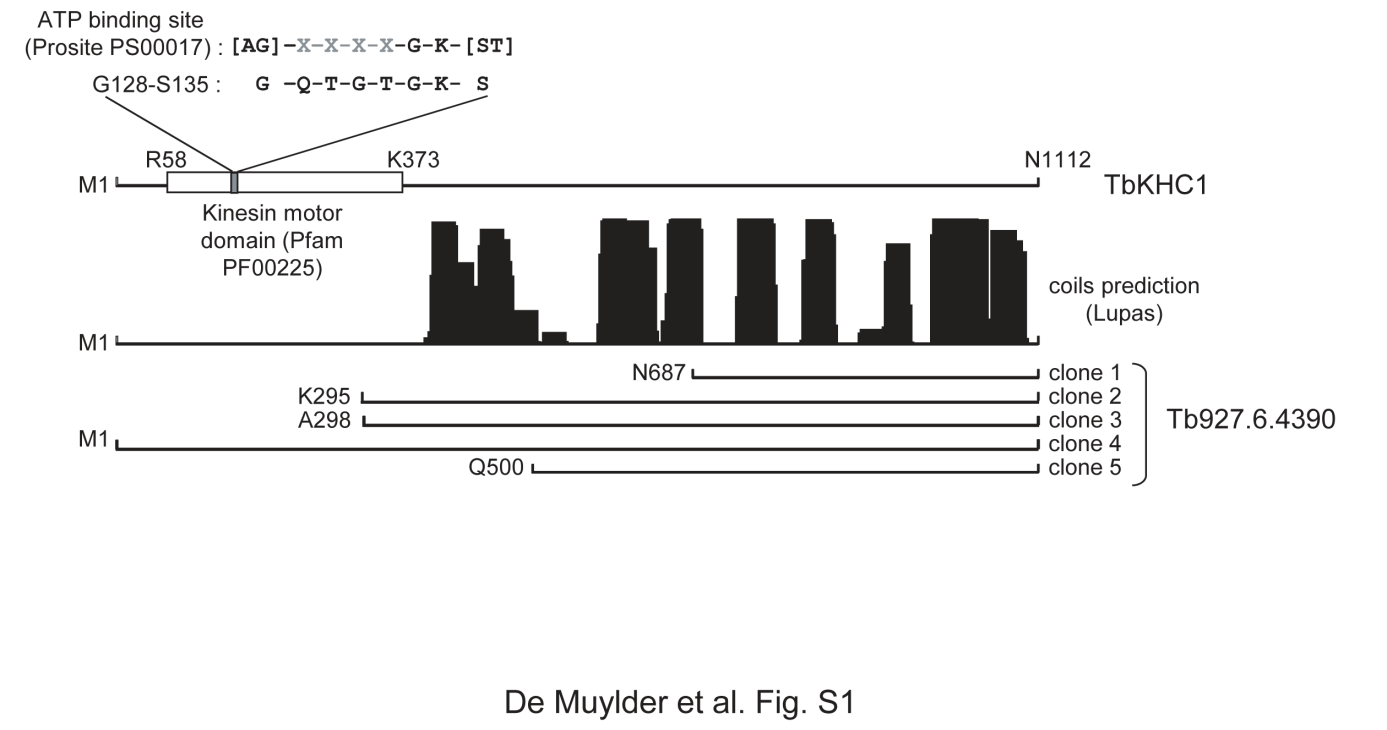
**

**
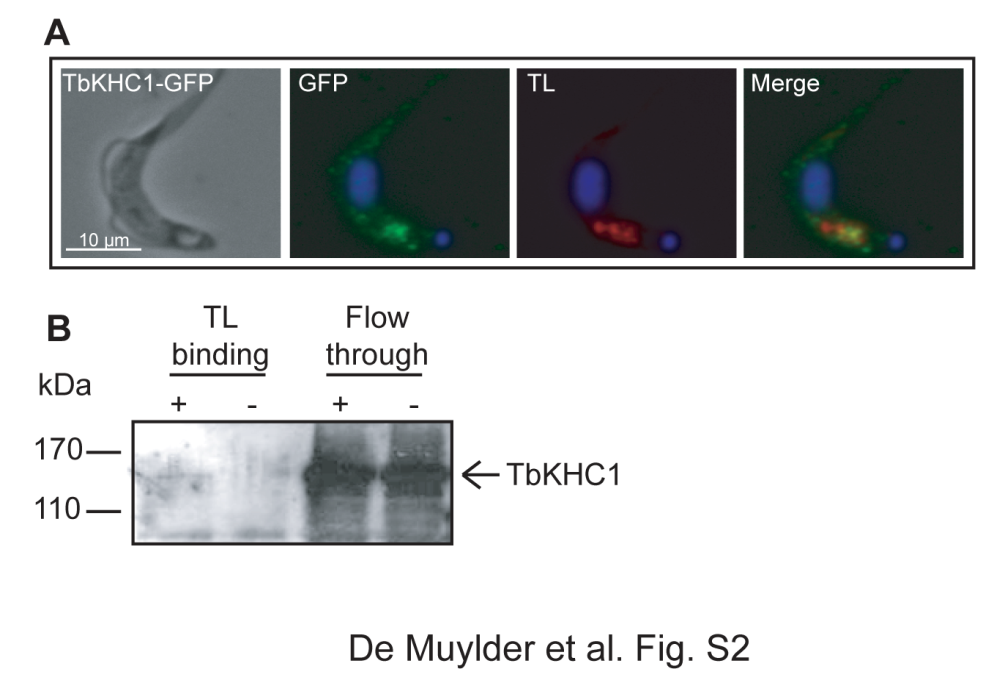
**

**
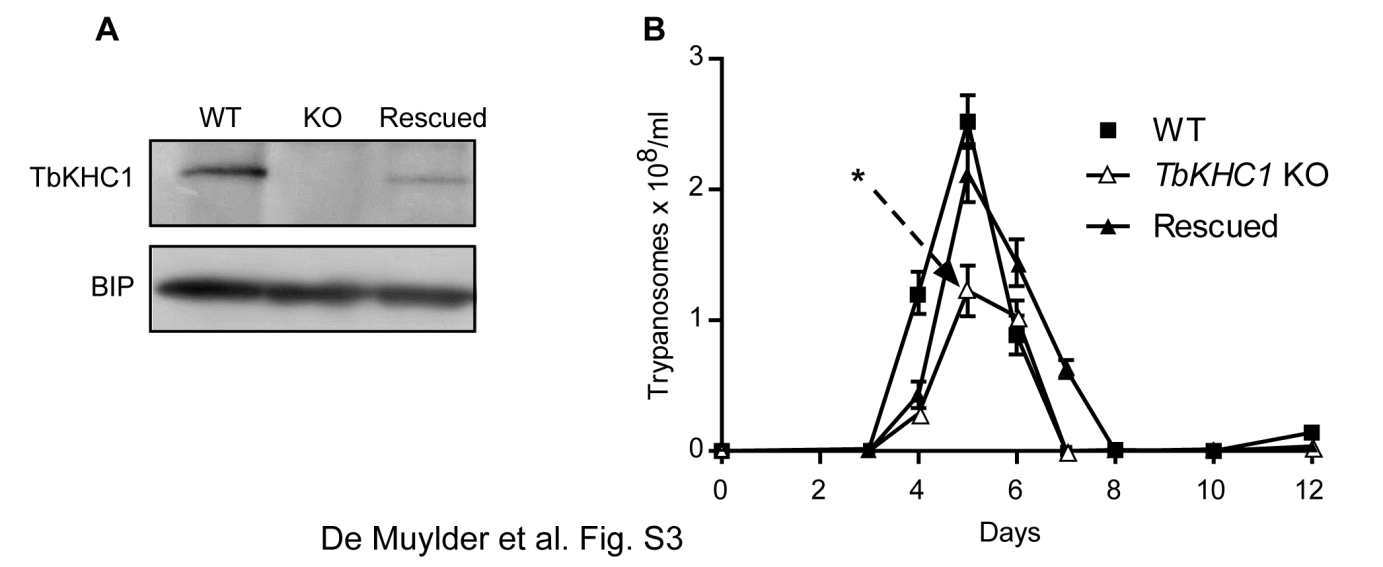
**

**
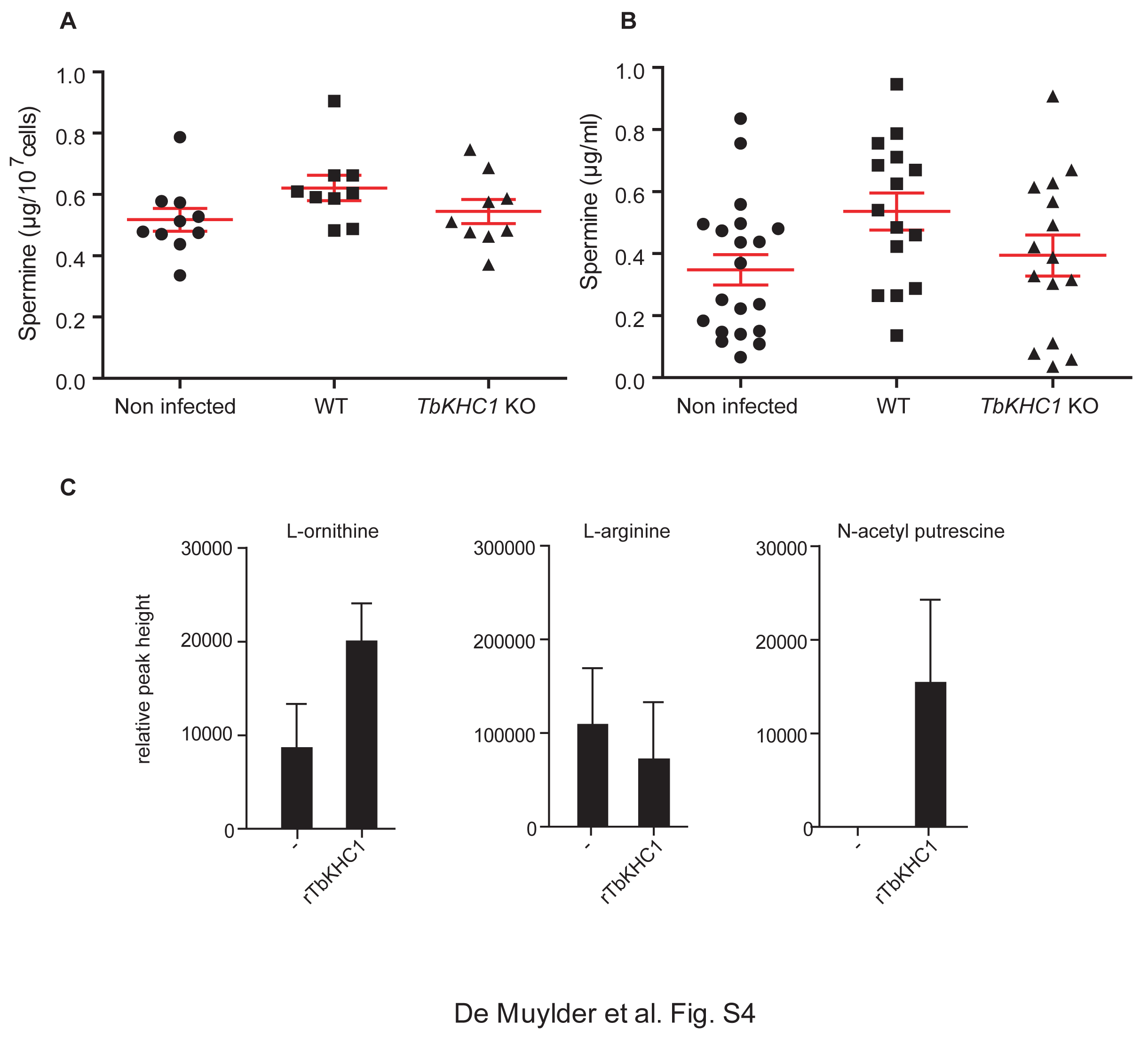
**

**
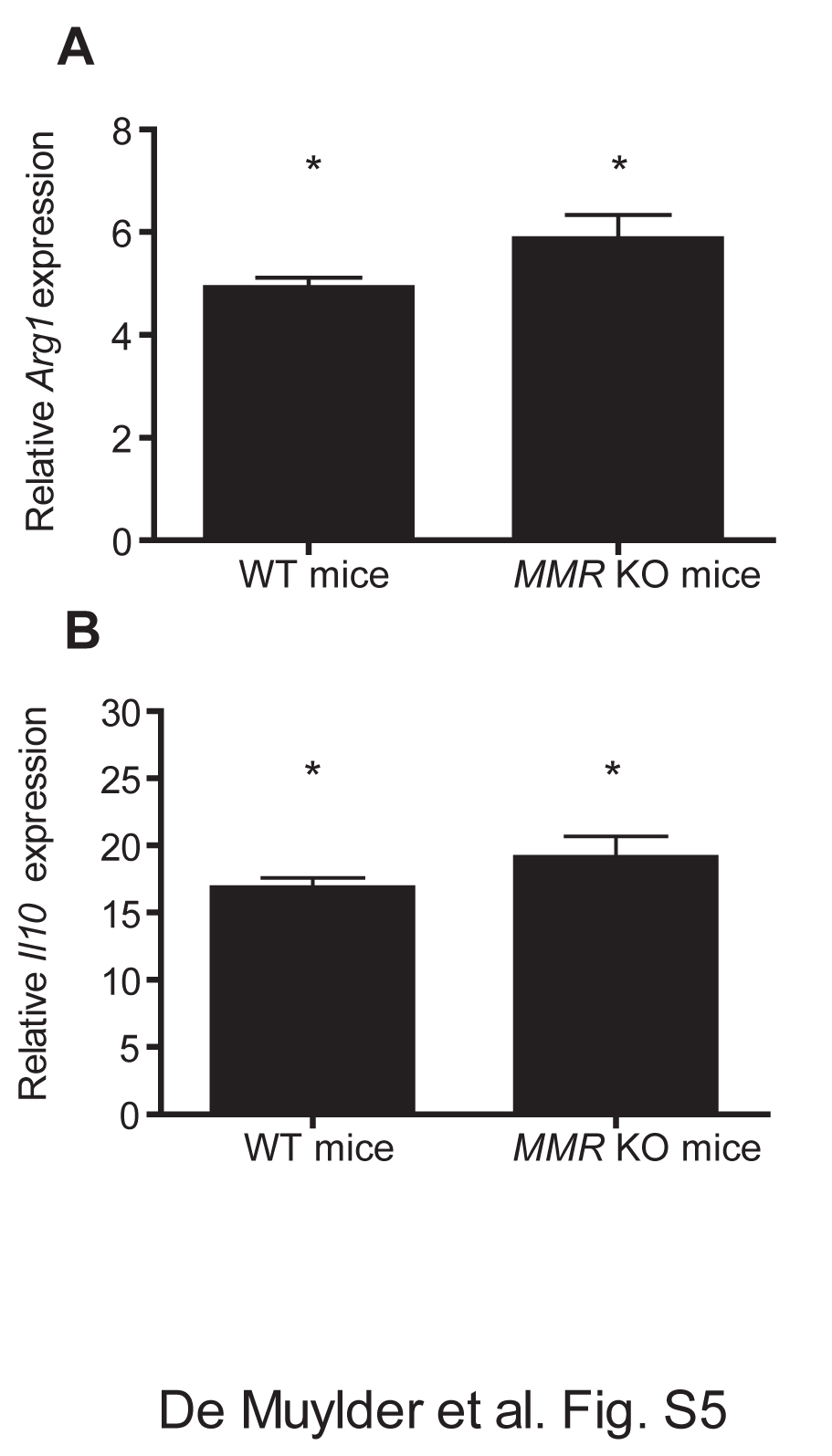
**

**
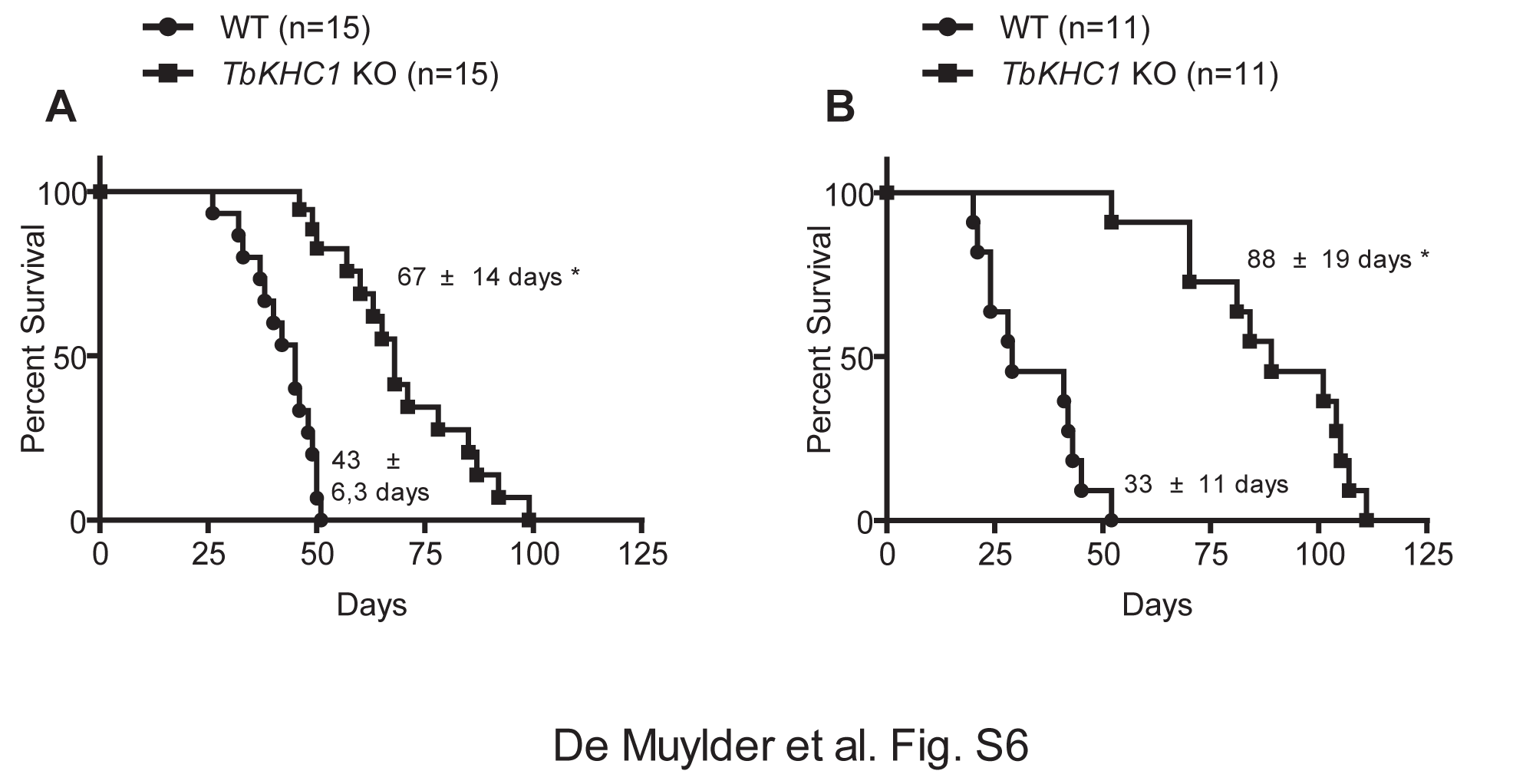
**

**
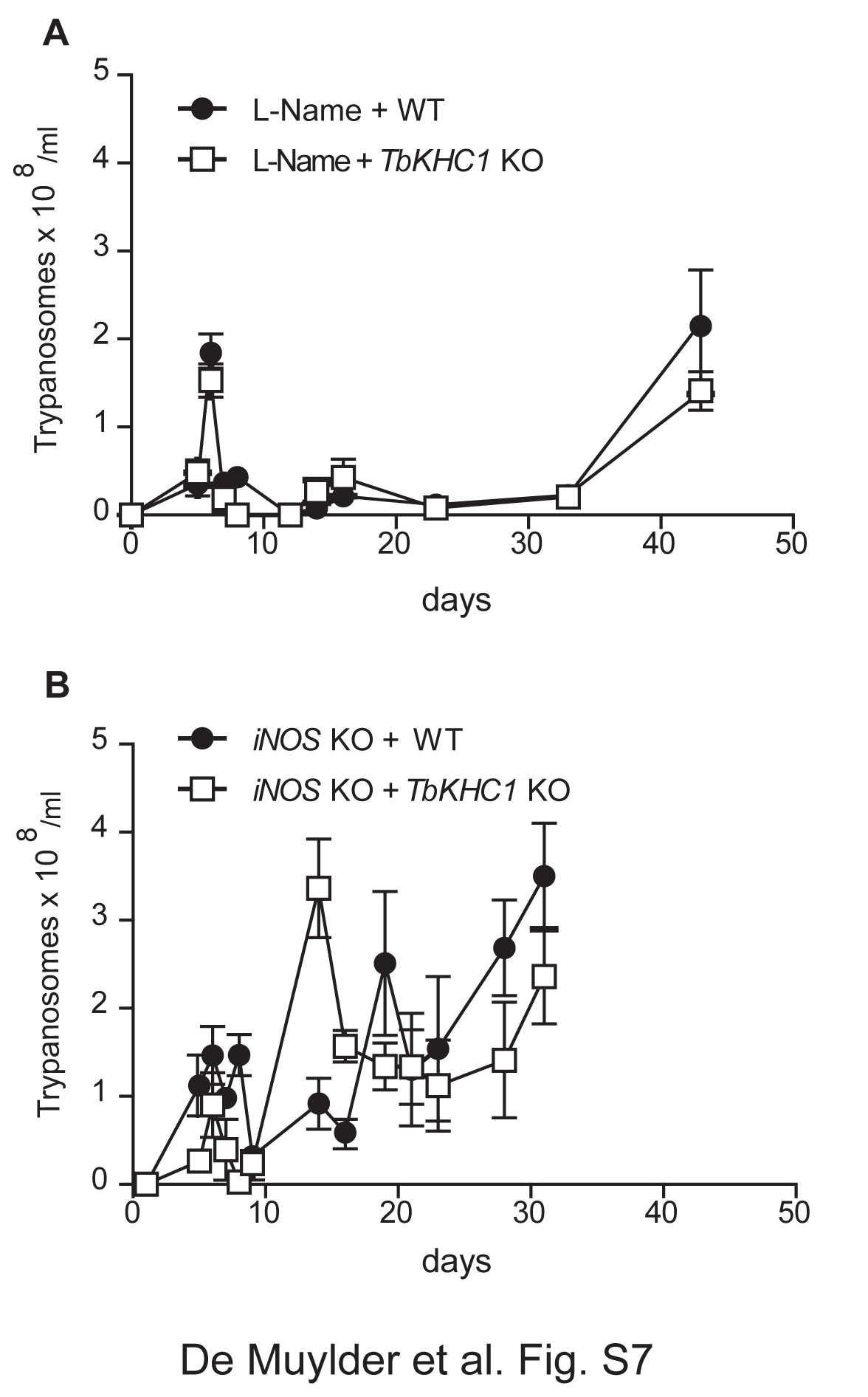
**

**
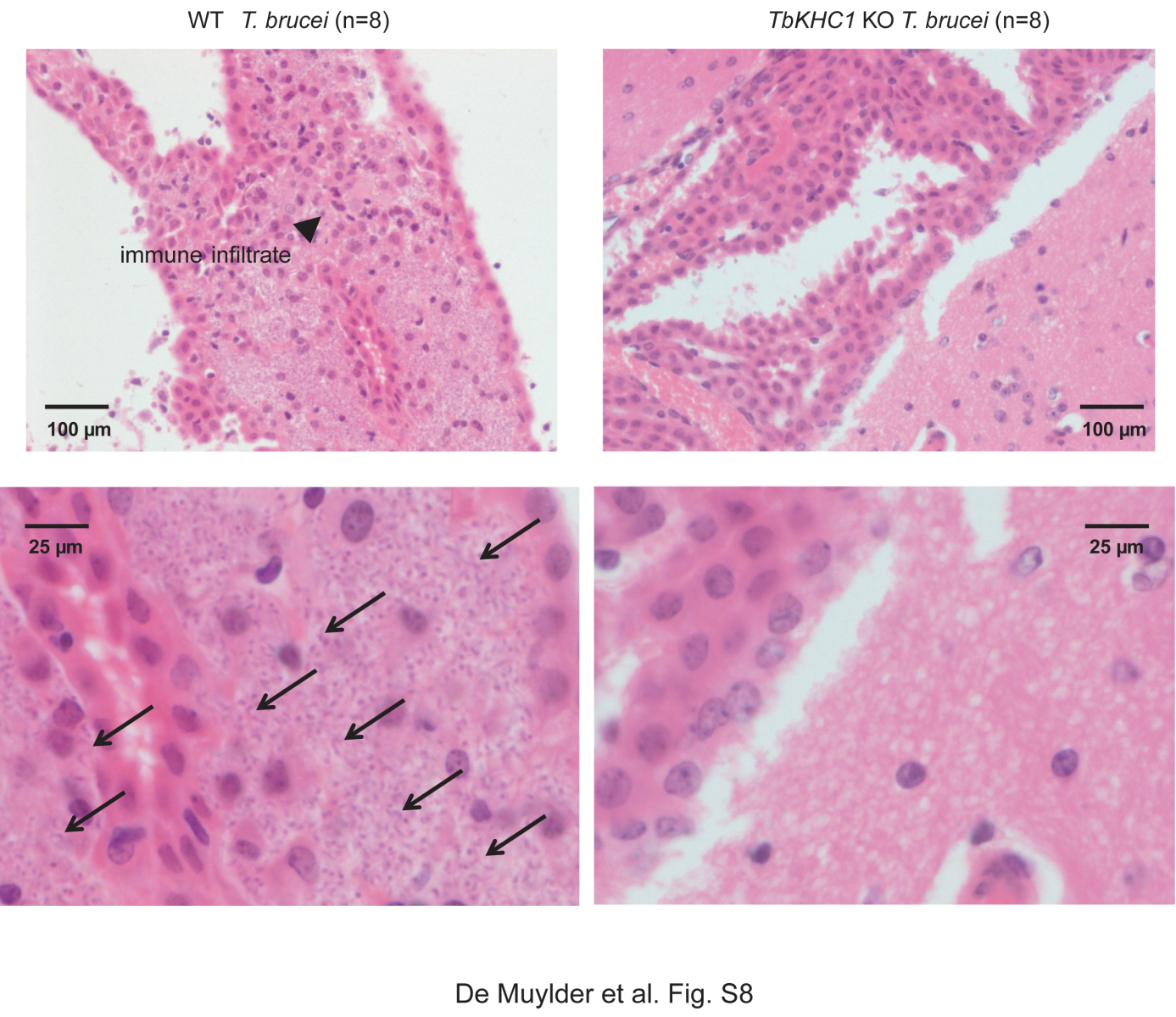
**

**Fig. S1. Schematic primary structure of *T. brucei* TbKHC1.** White and grey boxes represent the motor domain and the ATP binding site respectively. Coils predicted by the Lupas method are represented by black histograms. Sequences of the 5 positives cDNA clones encoded Tb927.6.4390 covering the entire (clone 4) or the C-terminal half (clone 1, 2, 3, 5) of TbKHC1 sequence. **Fig. S2. Localization of *T. brucei* TbKHC1.** (**A)** Co-localisation were performed on in situ tagged GFP-TbKHC1 cell line (green) and biotinylated tomato lectin (TL) (red). (**B)** Detection of TbKHC1 on WT bloodstream form trypanosome extracts bound or not (flow through) to tomato lectin (TL) after chromatography with (+) or without (-) competing chito-oligosaccharides. **Fig. S3. Characterization and growth pattern of *TbKHC1-*rescued *T. brucei*.** **(A)** Detection of TbKHC1 with anti-rTbKHC1 polyclonal antibody in whole extracts from WT, *TbKHC1* KO and *TbKHC1*-rescued parasites. BIP is the loading control. **(B)** Parasitaemia in WT mice infected with WT, *TbKHC1* KO or *TbKHC1-*rescued trypanosomes. Data are means ± SEM of 4 individual mice of one representative from 2 independent experiments. * p<0.05 comparing *TbKHC1 KO*- to *TbKHC1* rescued- or WT- infected mice*.* Similar results were obtained in 4 independent experiments using different rescued clones and mouse genetic backgrounds. Survival of mice infected with *TbKHC1*-rescued trypanosomes could not be determined due to transgene rearrangement during infection (loss of *TbKHC1* gene). **Fig. S4. Effect of *T. brucei* infection on metabolites involved in L-arginine pathway.** Spermine levels were determined **(A)** in blood and **(B)** spleen myeloid cell homogenate from WT mice at day 6 post infection with WT or *TbKHC1* KO parasites. Data are means ± SEM of 3 independent experiments pulled together. (C) Relative peak heights (y-axis) for L-ornithine, L-arginine and N-acetylputrescine from extracts of supernatants of myeloid cells either treated with rTbKHC1 or not (-) identified by LC-MS. **Fig. S5. Effect of rTbKHC1 on *Arg1* and *Il10* gene expression in *MMR* KO mice.** Myeloid cells from non infected WT or *MMR* KO mice were incubated with rTbKHC1. The relative gene expression of **(A)** *Arg1* and **(B)** *Il10* was determined. Data are means ± SEM of 3 individual mice of one representative from 3 independent experiments. * p<0.05 compared to non-stimulated cells. **Fig. S6. Effect of TbKHC1 on the survival of *T. brucei* infected mice.** Survival curves of WT C57Bl/6 mice infected with pleomorphic WT and TbKHC1 KO trypanosomes **(A)** i.p. or **(B)** through the bite of infected tsetse flies. Data are means ± SEM of 4 independent experiments pulled together. * p<0.05 comparing *TbKHC1* KO- and WT-infected mice. **Fig. S7. Effects of absence of *iNOS* gene activity on *T. brucei* parasitaemia.** WT and *TbKHC1* KO parasitaemias were monitored in **(A)** WT mice treated with L-NAME or **(B)** *iNOS* KO mice. Data are means ± SEM of 3 individual mice of one representative from 3 (A) or 2 (B) independent experiments. * p<0.05 mice infected with WT and *TbKHC1* KO parasites. **Fig. S8. Effects of TbKHC1 on cerebral injury in *T. brucei* infected mice.** Microscopic analysis (hematoxylin-eosin staining, magniﬁcation top pannels 40x, bottom pannels 160x) of brain choroid plexus from WT- and *TbKHC1* KO-infected mice at day 30 p.i. Immune cell infiltrates (►) and parasites (←) representative of 8 animals tested in 2 independent experiments are shown.
